# SAUR63 stimulates cell growth at the plasma membrane

**DOI:** 10.1371/journal.pgen.1010375

**Published:** 2022-09-19

**Authors:** Punita Nagpal, Paul H. Reeves, Jeh Haur Wong, Laia Armengot, Keun Chae, Nathaniel B. Rieveschl, Brendan Trinidad, Vala Davidsdottir, Prateek Jain, William M. Gray, Yvon Jaillais, Jason W. Reed

**Affiliations:** 1 Department of Biology, University of North Carolina at Chapel Hill, Chapel Hill, North Carolina, United States of America; 2 Laboratoire Reproduction et Développement des Plantes, Université de Lyon, ENS de Lyon, UCB Lyon 1, CNRS, INRAE, Lyon, France; 3 Department of Plant and Microbial Biology, University of Minnesota, St. Paul, Minnesota, United States of America; Wake Forest University, UNITED STATES

## Abstract

In plants, regulated cell expansion determines organ size and shape. Several members of the family of redundantly acting Small Auxin Up RNA (SAUR) proteins can stimulate plasma membrane (PM) H^+^-ATPase proton pumping activity by inhibiting PM-associated PP2C.D phosphatases, thereby increasing the PM electrochemical potential, acidifying the apoplast, and stimulating cell expansion. Similarly, *Arabidopsis thaliana* SAUR63 was able to increase growth of various organs, antagonize PP2C.D5 phosphatase, and increase H^+^-ATPase activity. Using a gain-of-function approach to bypass genetic redundancy, we dissected structural requirements for SAUR63 growth-promoting activity. The divergent N-terminal domain of SAUR63 has a predicted basic amphipathic α-helix and was able to drive partial PM association. Deletion of the N-terminal domain decreased PM association of a SAUR63 fusion protein, as well as decreasing protein level and eliminating growth-promoting activity. Conversely, forced PM association restored ability to promote H^+^-ATPase activity and cell expansion, indicating that SAUR63 is active when PM-associated. Lipid binding assays and perturbations of PM lipid composition indicate that the N-terminal domain can interact with PM anionic lipids. Mutations in the conserved SAUR domain also reduced PM association in root cells. Thus, both the N-terminal domain and the SAUR domain may cooperatively mediate the SAUR63 PM association required to promote growth.

## Author summary

Plant organs reach their final shape and size after substantial cell expansion. Proton pumps at the plasma membrane promote cell expansion by acidifying the cell wall to loosen it, and by increasing electrochemical potential across the plasma membrane for solute uptake that maintains intracellular turgor. Plasma-membrane-associated proteins tightly regulate proton pump activity, in order for organs to grow to an appropriate extent. We have studied requirements for activity of one such regulatory protein in the model plant Arabidopsis called SAUR63. This protein is made rapidly in response to plant growth hormones, and it increases proton pump activity to promote organ growth. These activities depend on its binding to anionic lipids in the plasma membrane, and forced plasma membrane association of SAUR63 can increase growth. Many proteins in the same family are found within Arabidopsis and in all land plants, and likely differ in their affinity for the plasma membrane or in other properties. Further studies of other family members may show how such proteins regulate growth under diverse physiological contexts.

## Introduction

Plant organs grow by extensive cell expansion after an initial period of cell division, and the geometry and extent of cell expansion determines mature plant form. Growth hormones can induce genes whose products promote cell expansion, including genes encoding cell wall modifying proteins and numerous *Small Auxin Up RNA* (*SAUR*) genes. *SAUR* genes are numerous in all land plants, and have evolved through extensive independent gene amplifications in multiple lineages [1, 2]. The model plant *Arabidopsis thaliana* has 79 *SAUR* genes. About half of Arabidopsis *SAUR* genes are induced rapidly by growth-promoting hormones or by environmental signals that stimulate growth, and many of the same genes are also repressed by stress treatments that impede growth [3–8].

Many *SAUR* genes have overlapping functions, and loss-of-function mutations in several growth-associated *SAUR* genes cause only subtle cell growth defects, such as affecting kinetics of growth responses during de-etiolation [4,7,9–12]. Gain-of-function approaches have helped to reveal SAUR protein activities despite apparent genetic redundancy. When overexpressed, several *SAUR* genes can increase cell and organ growth [10,13–16]. Moreover, several SAUR proteins have increased activity when expressed as fusion proteins. C-terminal GUS or GFP fusions to SAUR63, or N-terminal GFP or StrepII-tag fusions to SAUR19, increased cell expansion in growing hypocotyls, leaves, or flower organs [15–18]. Whereas SAUR63:3xHA fusion protein or untagged SAUR19 had *in vivo* half-lives of about 10 minutes and did not detectably affect cell growth, the GFP:SAUR19 and SAUR63:GFP fusion proteins each had much longer half-lives [15,16]. Untagged SAUR19 as well as the SAUR19 and SAUR63 fusion proteins also localized partly to the plasma membrane (PM).

SAUR proteins from several clades can inhibit activity of the PP2C.D family of phosphatases, and thereby stimulate activity of the PM H^+^-ATPase [7,12,19–23]. In a process called acid growth, the PM H^+^-ATPase acidifies the apoplastic space to promote cell wall loosening, and also increases the membrane electrochemical potential to drive ion uptake, thereby maintaining intracellular osmotic potential and turgor [24,25]. H^+^-ATPase activity level is determined by a balance of activation by phosphorylation at the penultimate residue (Thr^947^ in the AHA2 isoform), through activity of multiple Trans-Membrane Kinases (TMKs) and possibly other kinases [26,27], and inactivation through dephosphorylation by PP2C.D phosphatases [19]. A subset of the PP2C.D phosphatases localize to the plasma membrane [28]. The plasma membrane thus emerges as a potentially important location of cell growth control, where SAUR and PP2C.D proteins may interact to regulate the H^+^-ATPase and possibly other targets. How SAUR and PP2C.D proteins localize to the PM is not known.

Other *SAUR* genes that are not induced in growing tissues regulate aspects of physiology rather than cell expansion. For example, the guard-cell-expressed *SAUR56* and *SAUR60* genes promote stomatal opening [22]; and SAUR36 and SAUR49 proteins can promote leaf senescence [21,29]. Gain-of-function SAUR19 and SAUR63 fusion proteins caused constitutively open stomata when expressed behind the *P*_*35S*_ promoter [22], suggesting that SAURs 19, 56, 60, and 63 have similar biochemical activities. In guard cells and senescing leaves, SAUR proteins likely inhibit PP2C.D phosphatases and thereby promote H^+^-ATPase activity [21,22].

SAUR proteins have a conserved central domain of about 70 amino acids that defines membership in the family [6,30]. In contrast, the N- and C-terminal domains of Arabidopsis SAUR proteins vary in length (from 18 to 85 amino acids for the N-terminal domain) and differ substantially among different subgroups. The SAUR63 protein and its close relatives have N- and C-terminal extensions of 30–40 amino acids that are conserved only within the SAUR63 subclade. The questions arise as to whether SAUR63 functions similarly to other SAUR proteins, and whether these extensions confer unique aspects of SAUR63 clade function.

We have used growth assays and variant gain-of-function SAUR63 fusion proteins to explore how SAUR63 localizes to the PM, and the importance of this localization. We find that both the N-terminal and the SAUR domains of SAUR63 promote PM localization and protein stability. Growth phenotypes of plants expressing SAUR63 derivatives with altered localization reveal that PM localization is essential for SAUR63 to stimulate cell growth. The N-terminal domain has a predicted amphipathic α-helix that can associate with PM anionic phospholipids, thereby concentrating SAUR63 close to its regulatory targets. Portions of the SAUR domain are also required for robust PM localization.

## Results

### Overexpression of SAUR63:X fusion proteins increases growth in shoot organs

SAUR63 is a member of a clade of nine Arabidopsis SAUR proteins that radiated fairly recently in evolution [6,30], most of which are expressed similarly in growing shoot tissues [5,7,15,17]. We used two rounds of CRISPR/Cas9-mediated genome editing to create a plant with mutations in all nine of these genes (S1A Fig). The eight clustered genes on chromosome 1 were either deleted or had a frameshift in the coding sequence, and these mutations therefore likely eliminate protein function (S1A and S1F Fig). The *saur75-1* mutation on chromosome 5 had a predicted in-frame deletion of amino acids 70–82 (QELLKISEEEFGL) in the center of the SAUR domain (S1A and S1F Fig). Although many of these residues are conserved among SAUR proteins [6], replacement of stretches of six amino acids corresponding to portions of this segment of SAUR63 (in NAAIRS mutants *m12* and *m13* below) did not eliminate function, so further work would be needed to assess whether this deletion eliminates SAUR75 activity. Hypocotyl and cotyledon growth of the resulting nonuple mutant was indistinguishable from growth of wild-type seedlings under our standard conditions (S1B–S1E Fig). While more refined assays might reveal growth defects, these are likely to be subtle enough to complicate structure-function studies by phenotypic rescue with SAUR63 variants.

**Fig 1:**
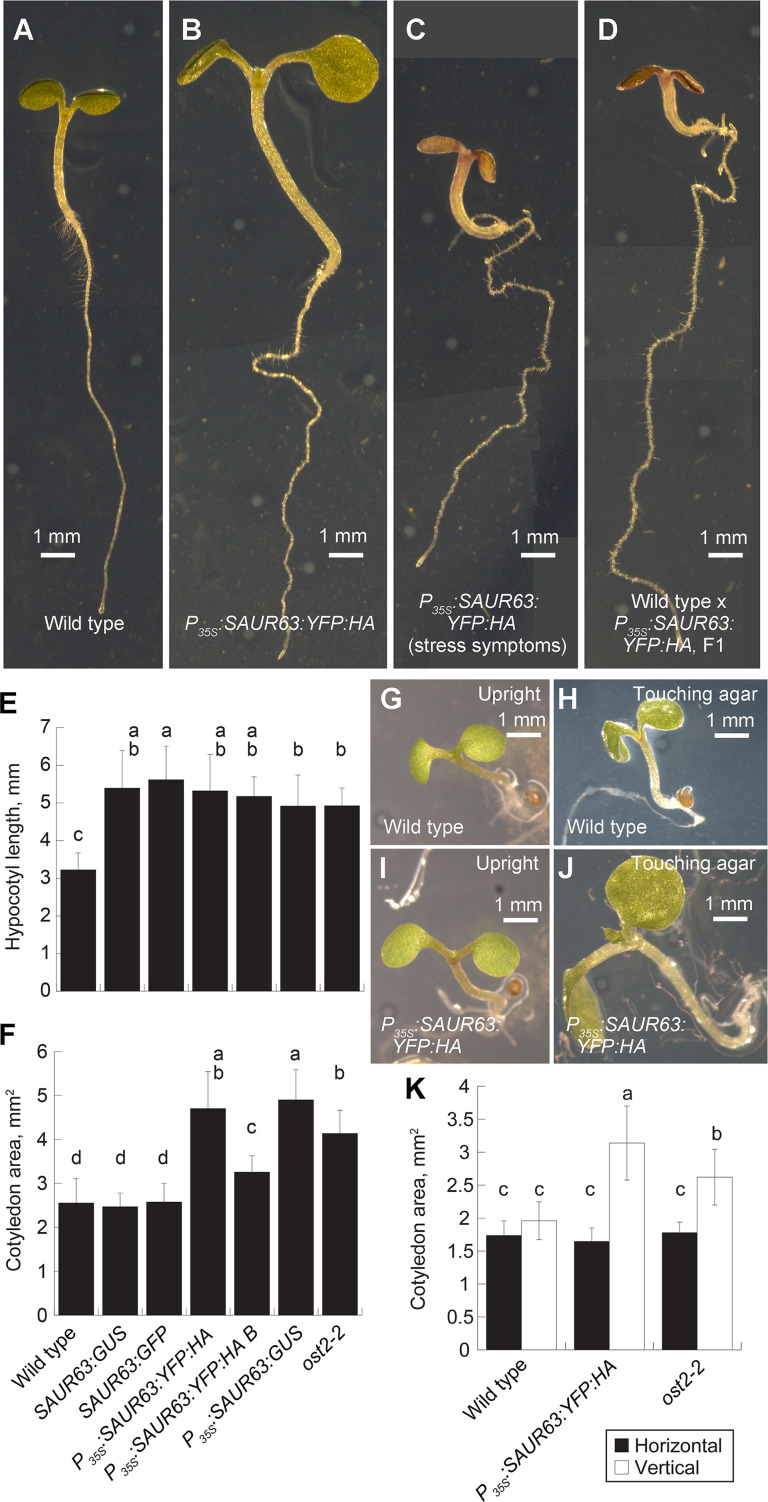
Hypocotyl and cotyledon phenotypes of plants expressing SAUR63:X fusion proteins. **A-D)** Photographs of 5-day-old seedlings of indicated genotypes. Seedlings were grown in long days in presence of sucrose to allow root growth of all genotypes. For the F1 seedling shown in D, wild type was the female parent. Scale bars, 1 mm. **E)** Hypocotyl lengths of seedlings of indicated genotypes grown for 4d in short days on 0.5x MS medium. **F)** Cotyledon areas of seedlings of indicated genotypes grown for 6d in long days on 1x MS 1% sucrose medium. Genotype designations in F also refer to panel E. In E and F, data for two independent *P*_*35S*_:*SAUR63*:*YFP*:*HA* lines are shown. **G-J)** Appearance of individual wild-type (G,H) or *P*_*35S*_:*SAUR63*:*YFP*:*HA* (I,J) seedlings grown on horizontally oriented plates for 6d in long days in the presence of sucrose. Shown are seedlings that grew upright (G,I) or grew recumbently with cotyledons touching the agar (H,J). Scale bar, 1 mm. **K)** Cotyledon areas of seedlings grown on either horizontally or vertically oriented agar plates with sucrose for 6d in long days. For horizontally oriented plates, only seedlings that grew upright with cotyledons not touching the agar were used for the cotyledon area calculation. Graphs show means ± s.d. Letters in graphs indicate statistical classes based on Tukey’s Honestly Significant Difference test. n, from left to right: panel E: 51, 29, 21, 22, 25, 22, 24; panel F: 26, 19, 11, 19, 16, 18; panel K: 32, 23, 24, 15, 22, 23, 17.

To probe structural requirements for SAUR63 function, we therefore used a gain-of-function approach. *SAUR63*:*GUS* and *SAUR63*:*GFP* plants expressing stabilized fusion proteins behind the native promoter have elongated hypocotyls [15]. Likewise, *P*_*35S*_:*SAUR63*:*GUS* and *P*_*35S*_:*SAUR63*:*YFP*:*HA* seedlings had elongated hypocotyls arising from increased cell expansion in the light (Figs 1A, 1B, 1E and S2 and S3G and S3H). Hypocotyls of dark-grown *P*_*35S*_:*SAUR63*:*YFP*:*HA* seedlings were of similar length to those of wild-type seedlings, but were more tortuous (not straight) in aspect (S3A and S3B Fig). Unlike *SAUR63*:*GUS* and *SAUR63*:*GFP* seedlings, *P*_*35S*_:*SAUR63*:*YFP*:*HA* and *P*_*35S*_:*SAUR63*:*GUS* seedlings also had markedly increased cotyledon growth (Fig 1F). Excess cotyledon growth depended on the presence of exogenous sucrose and on contact with the agar surface (Figs 1G–1K and S4A), suggesting that the cotyledons may have taken up sucrose directly from the medium, as found previously [31]. Finally, roots of *P*_*35S*_:*SAUR63*:*GUS* and *P*_*35S*_:*SAUR63*:*YFP*:*HA* lines grew with a more tortuous trajectory than did wild-type roots, but grew slowly in the absence of exogenous sucrose (Figs 1A and 1B and S3C–S3F). Some *P*_*35S*_:*SAUR63*:*YFP*:*HA* seed batches produced a fraction of small purple seedlings (Fig 1C). F1 plants arising after fertilizing wild-type gynoecia with *P*_*35S*_:*SAUR63*:*YFP*:*HA* pollen were also often small and purple (Fig 1D), suggesting that this phenotype depends on the zygotic genotype and that increased SAUR63 activity may sometimes activate stress responses during embryo or seedling development. These seedling growth phenotypes served as convenient measurable assays to probe activity of SAUR63 variants in experiments described below.

### SAUR63 can antagonize effects of PP2C.D phosphatases

**Fig 2:**
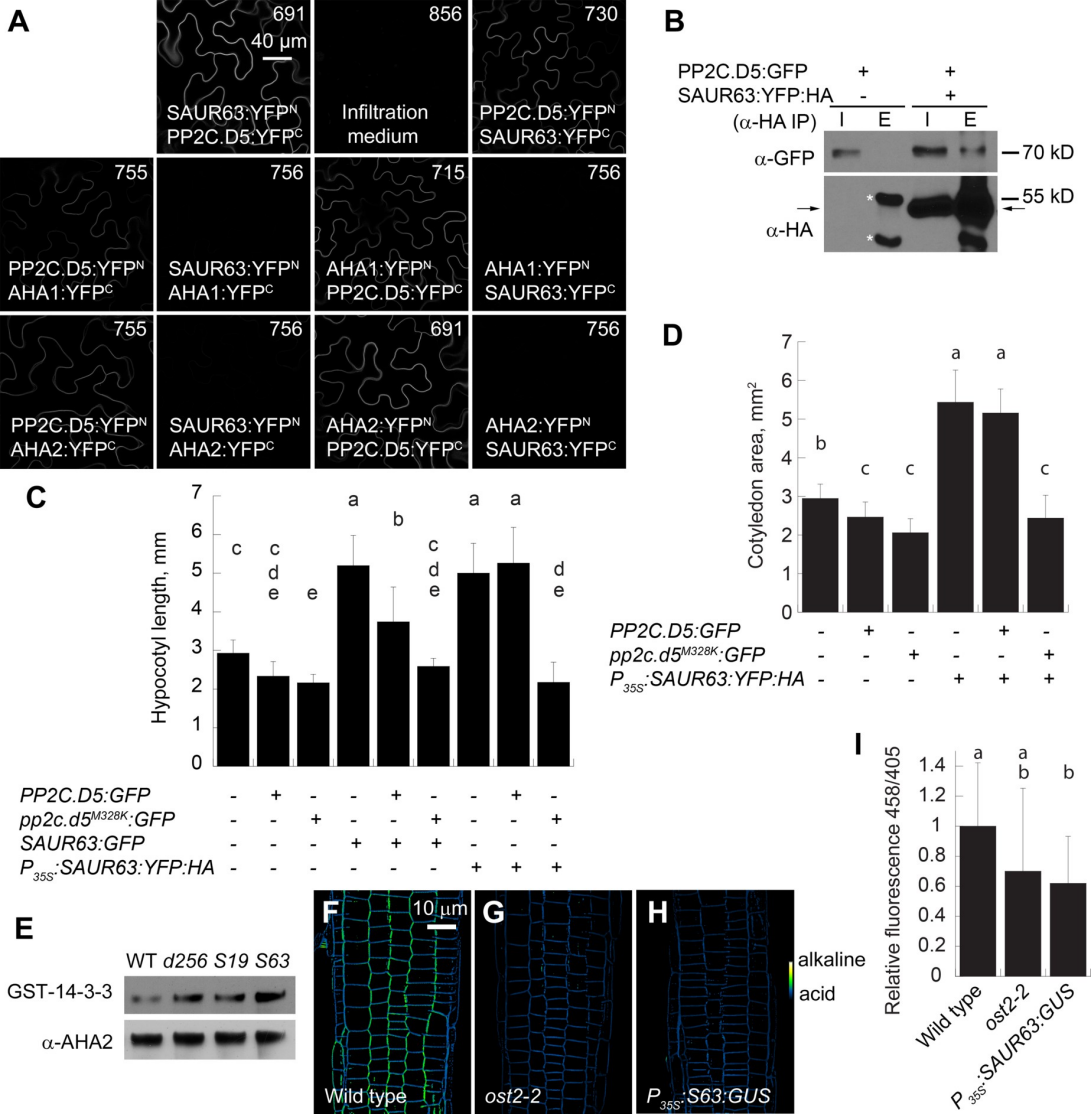
SAUR63 interactions with PP2C.D proteins. **A)** Bimolecular fluorescence complementation assays of PP2C.D5 with SAUR63. Reconstituted yellow fluorescence from the N- and C-terminal portions of YFP (YFP^N^ and YFP^C^) indicates protein-protein interaction. AHA1 and AHA2 fusions, which interact with PP2C.D5, were used as controls. Scale bar, 40 μm. Numbers in each panel indicate gain setting used for confocal image acquisition. SAUR63:YFP:HA (with complete YFP) localized to the cell periphery as shown in S8A Fig. **B)** Co-immunoprecipitation of PP2C.D5:GFP protein with immunoprecipitated SAUR63:YFP:HA using α-HA antibody, from microsomes of pooled F2 seedlings. I, Input; E: Eluate. The upper blot shows co-immunoprecipitated PP2C.D5:GFP fusion protein detected with α-GFP antibody. In the lower blot, the arrow indicates the immunoprecipitated SAUR63:YFP:HA protein detcted with α-HA antibody. Asterisks indicate the heavy and light chains of the IgG protein used for immunoprecipitation, which partially obscure the SAUR63:YFP:HA protein in the eluate lane. Replicate experiments gave comparable results. **C)** Hypocotyl lengths of seedlings of indicated genotypes grown for 4d in short days in the absence of sucrose. n, from left to right: 28, 24, 24, 26, 25, 26, 25, 24, 20. A replicate experiment gave similar results. S5B Fig shows the complete dataset from which this data was taken. **D)** Cotyledon areas of seedlings grown on vertically oriented agar plates with sucrose for 6d in long days. n, from left to right: 27, 22, 24, 21, 17, 17. A replicate experiment gave similar results. In C and D, seedlings used were F1 progeny of crosses of transgenic lines with each other or with wild-type Columbia, and were hemizygous for the indicated transgene(s). **E)** Detection of Thr^947^-phosphorylated H^+^-ATPase by a 14-3-3 protein overlay assay in proteins extracted from microsomes of wild-type, *pp2c*.*d2 pp2c*.*d5 pp2c*.*d6* triple mutant (*d256*), *P*_*35S*_:*GFP*:*SAUR19*, and *P*_*35S*_:*SAUR63*:*YFP*:*HA* shoots. Seedlings were grown in light for 4 days. The α-AHA2 blot shows overall levels of H^+^-ATPase in each genotype. **F-H)** Images of 8-Hydroxypyrene-1,3,6-trisulfonic acid (HPTS) staining in indicated genotypes. Images show ratio of 458 nm/405 nm excitation. The color scale at right in panel H indicates the color range used to depict higher or lower ratios corresponding to more alkaline or acid pH. Scale bar, 10 μm. **I)** Quantitation of HPTS staining. Data are pooled from measurements of three different experiments, with each 458 nm/405 nm signal ratio calculation normalized to the corresponding wild-type ratio in the same experiment. n, from left to right: 17, 14, 16. Graphs in panels C, D, and I show means ± s.d. Letters on graphs indicate statistical classes based on Tukey’s Honestly Significant Difference test.

Although SAUR19 and SAUR63 are in distinct clades, the *P*_*35S*_:*SAUR63*:*YFP*:*HA* phenotypes closely resemble those of *P*_*35S*_:*GFP*:*SAUR19* plants overexpressing stabilized GFP:SAUR19 fusion protein [16]. The dominant *ost2-2* (*open-stomata2*) mutation in *AHA1* increases its proton-pumping activity independently of SAUR and PP2C.D action, and also increased hypocotyl and cotyledon growth (Figs 1E and 1F and S2 and S3I) [19,32]. As SAUR19 increases H^+^-ATPase activity by inhibiting PP2C.D phosphatases, we tested whether SAUR63 might act in this manner. As experiments described below indicate that SAUR63 acts at the PM, we focused our experiments on PP2C.D5, which is primarily PM-localized and for which transgenic fusion protein lines were available [20,28]. Indeed, in bimolecular fluorescence complementation (BiFC) assays in transiently transformed *Nicotiana benthamiana* leaf cells, SAUR63 interacted with PP2C.D5 at the cell periphery (Fig 2A). Little or no BiFC signal was detected in assays including SAUR63 with AHA1 and AHA2 H^+^-ATPase proteins, which are integral PM proteins and could each interact robustly with PP2C.D5 (Fig 2A) [20,28]. In microsomal fractions from seedlings expressing PP2C.D5:GFP protein behind its native promoter and SAUR63:YFP:HA behind the *P*_*35S*_ promoter, α-HA antibody immunoprecipitated PP2C.D5:GFP along with SAUR63:YFP:HA, whereas no PP2C.D5:GFP was immunoprecipitated in plants lacking SAUR63:YFP:HA (Fig 2B). These assays indicate that SAUR63 is able to interact with PP2C.D5.

We obtained only insoluble SAUR63 recombinant proteins after heterologous expression in *E*. *coli*, precluding direct assay of SAUR63 effects on PP2C.D5 phosphatase activity. Instead, we probed their *in vivo* functional interactions by genetic epistasis experiments. The *PP2C*.*D5*:*GFP* and *pp2c*.*d5*^*M328K*^:*GFP* transgenes each decrease hypocotyl and cotyledon growth, and the M328K mutation also renders the PP2C.D5 protein immune to SAUR19 inhibition ](S5A Fig). We measured hypocotyl growth in the F1 progeny of gain-of-function *SAUR63* plants crossed with *PP2C*.*D5*:*GFP* and *pp2c*.*d5*^*M328K*^:*GFP* plants. *SAUR63*:*GUS* and *SAUR63*:*GFP* combinations with *PP2C*.*D5*:*GFP* (each expressed behind the corresponding native promoter) had hypocotyl lengths intermediate between those of the two parent hemizygotes (Figs 2C and S5B). The stronger *P*_*35S*_:*SAUR63*:*YFP*:*HA* transgenes were epistatic to the *PP2C*.*D5*:*GFP* transgene, conferring elongated hypocotyls similar to the effect of *P*_*35S*_:*SAUR63*:*YFP*:*HA* alone (Figs 2C and S5B), possibly reflecting a higher expression level or broader expression domain of *P*_*35S*_ compared to *P*_*SAUR63*_. Similarly, a strong *P*_*35S*_:*SAUR63*:*YFP*:*HA* line caused equally large cotyledons in wild-type and *PP2C*.*D5*:*GFP* backgrounds (Fig 2D). These results indicate that SAUR63 and PP2C.D5 can act antagonistically to regulate hypocotyl elongation and cotyledon growth, with the balance between SAUR63 and PP2C.D5 determining the extent of growth. In contrast, the *pp2c*.*d5*^*M328K*^:*GFP* gene, expressing a SAUR-immune PP2C.D5 mutant protein, suppressed the hypocotyl growth-promoting effects of SAUR63 transgenes expressed behind either native or *P*_*35S*_ promoters, and the enlarged cotyledon growth caused by strong *P*_*35S*_:*SAUR63*:*YFP*:*HA* expression (Figs 2C and 2D and S5B). These results suggest that PP2C.D5 acts downstream of SAUR63, similarly to its relation with SAUR19 [20].

Consistent with the model that high SAUR63 activity maintains the PM H^+^-ATPase in an active state, estradiol-induced *P*_*EST*_:*SAUR63*:*CerFP*:*HA* [22] and *P*_*35S*_:*SAUR63*:*YFP*:*HA* seedlings were hypersensitive to the toxic cation Li^+^ in the growth medium (S5C and S5D Fig). In both loss- and gain-of-function mutants, Li^+^ sensitivity correlates with PM H^+^-ATPase activity, likely because the PM H^+^-ATPase creates the plasma membrane potential that then drives toxic cation uptake [19,33,34]. Moreover, a 14-3-3 protein far-western blot assay showed that 4-day-old *P*_*35S*_:*SAUR63*:*YFP*:*HA*, *pp2c*.*d2/5/6* triple mutant, and *P*_*35S*_:*GFP*:*SAUR19* seedlings each had a higher level of Thr^947^-phosphorylated H^+^-ATPase than did wild-type seedlings (Fig 2E). Lastly, using the extracellular ratiometric pH indicator 8-Hydroxypyrene-1,3,6-trisulfonic acid (HPTS), we found slightly lower pH in the apoplastic space surrounding root meristem cells of *P*_*35S*_:*SAUR63*:*GUS* and *P*_*35S*_:*SAUR63*:*YFP*:*HA* than in wild-type roots, although the variability was such that the difference from wild type was statistically significant only for the *P*_*35S*_:*SAUR63*:*GUS* roots (Figs 2F–2I and S5E). These interaction assays and phenotypes suggest that, similarly to several other SAUR proteins, SAUR63 likely inhibits PP2C.D phosphatase activity to derepress the proton pump [7,12,19–22].

### The N-terminal domain promotes SAUR63 plasma membrane localization and stability

**Fig 3:**
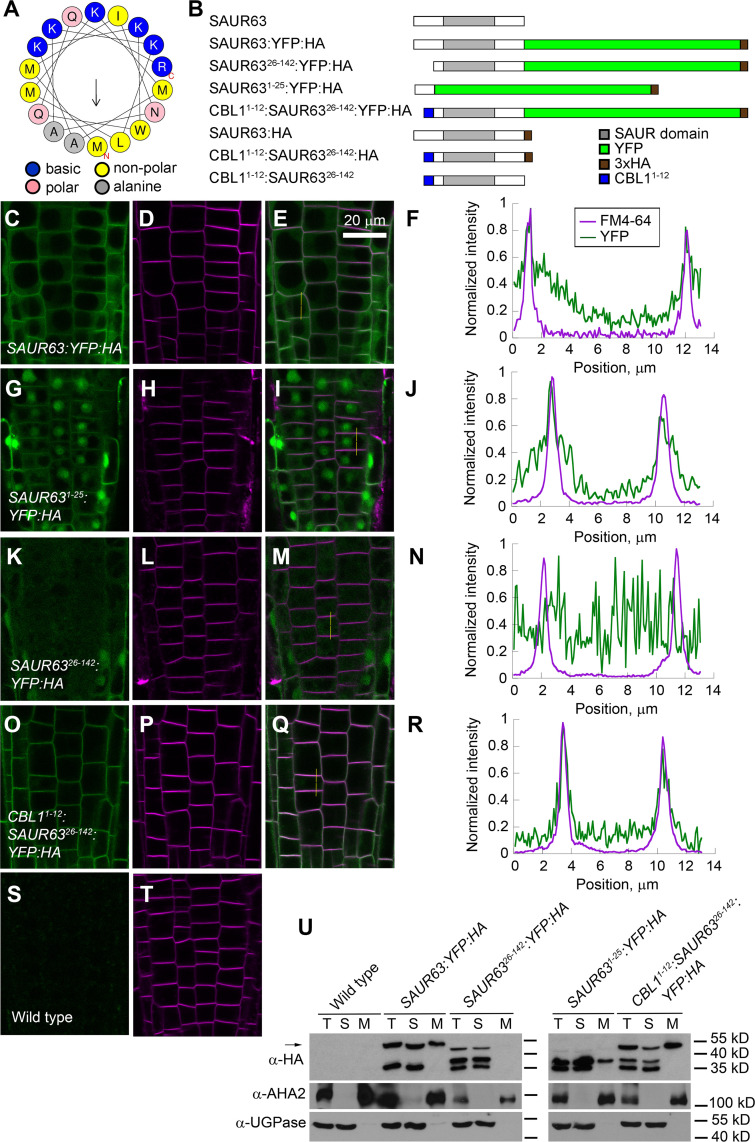
Effects of N-terminal domain manipulations on SAUR63 fusion protein localization. **A)** Helical wheel projection of SAUR63 N-terminal 18 amino acids. Arrow indicates direction of hydrophobic moment. **B)** Diagram of SAUR63 and various fusion proteins overexpressed in this study. Gray bar, SAUR domain; Green, YFP; Brown, HA epitope tag; Blue, Myristoylation and palmitoylation signal from CBL1. **C-R)** Visualization of SAUR63:YFP:HA fusion proteins in root meristem epidermal cells. **C-F)** SAUR63:YFP:HA. **G-J)** SAUR63^1-25^:YFP:HA. **K-N)** SAUR63^26-142^:YFP:HA. **O-R)** CBL1^1-12^:SAUR63^26-142^:YFP:HA. **S,T)** Wild-type Columbia. Shown are YFP (green, C,G,K,O,S), FM4-64 membrane staining (magenta, panels D,H,L,P,T), both channels together (E,I,M,Q), and quantitation of fluorescence intensity signal along the vertical yellow lines in panels E,I,M,Q, scaled to the maximum signal along the line (panels F,J,N,R). Image color channel brightnesses were adjusted for visibility. In S, the signal was enhanced digitally by the same degree as in panel K to show the level of background fluorescence. Scale bar, 20 μm. **U)** SAUR63:YFP:HA, SAUR63^26-142^:YFP:HA, SAUR63^1-25^:YFP:HA, and CBL1^1-12^:SAUR63^26-142^:YFP:HA fusion proteins, detected by western blots in total (T), soluble (S) and microsomal (M) protein fractions. α-HA detects SAUR63:YFP:HA fusion protein variants; α-AHA2 detects a membrane protein; α-UGPase detects soluble proteins. The arrow indicates the position of full-sized fusion protein. Positions of protein size markers are indicated. S9 Fig shows blots of additional lines.

Amino acid sequences N-terminal to the conserved SAUR domain are generally quite divergent except within subclades of the SAUR protein family [6]. SAUR63 has 38 amino acids N-terminal to the conserved SAUR domain. This sequence is enriched in both basic and hydrophobic amino acids, having a net charge of +10 (+9 in the first 25 amino acids). The Basic Hydrophobic domain (BH) algorithm [35] gave the N-terminal domain of SAUR63 an integrated BH score (summed for all 12-amino-acid segments above a threshold score of 0.6) of 4.21. The PsiPred 4.0 algorithm predicts that amino acids 5–22 can form an α-helix [36,37], whereas the Heliquest algorithm indicates that amino acids 1–18 have the potential to form an α-helix with a predicted hydrophobic moment of 0.39 (Fig 3A and S1 Table) [38]. The BH and Heliquest scores are each above standard confidence thresholds for these algorithms. Similarly, the AlphaFold 2.0 algorithm predicts that amino acids 5–22 of SAUR63 form an α-helix [39](see https://alphafold.ebi.ac.uk/entry/F4I1H5). These predictions suggest that the N-terminal 25 amino acids of SAUR63 encompass an amphipathic α-helix with basic and hydrophobic faces, a feature that in many proteins can mediate membrane association [35,40–43]. Other members of the SAUR63 clade (SAUR61-SAUR68, SAUR75) have N-terminal domains of similar length and with similar predicted properties (S1 Table).

We used variant SAUR63:YFP:HA fusion proteins to explore whether the N-terminal domain affects localization (Fig 3B). Root meristem cells have small vacuoles, such that WAVE line markers of PM, cytoplasm, and tonoplast subcellular compartments are easily distinguishable by confocal microscopy [44](S6I–S6T Fig). The fusion protein in *P*_*35S*_:*SAUR63*:*YFP*:*HA* plants was detected most strongly at the cell periphery in epidermal cells of the root meristem zone (Figs 3C–3E and S6A–S6D). In these cells, coincidence with signal from FM4-64 membrane stain suggests that this signal reflects PM association (at least within the resolution of the confocal images, Figs 3F and S6D). In addition to PM localization, weaker fluorescence was also visible in the cytoplasm and in the nucleus. In epidermal cells of cotyledons, the SAUR63:YFP:HA fusion protein also localized to the cell periphery, consistent with PM localization, as well as to nuclei (S7A Fig). However, because of the narrow band of cytoplasm in expanded cotyledon cells, control fusion proteins localizing to PM and cytoplasm appeared to localize very similarly and could not be distinguished easily in these cells (S7B and S7C Fig). A tonoplast marker was also peripherally localized in cotyledon cells, but could be seen in two stripes corresponding to adjacent cells, thus distinguishing tonoplast from PM and cytoplasm markers (S7D Fig). As we could not reliably distinguish PM from cytoplasm in cotyledon cells, we mainly used root meristem zone cells to visualize localization of variant SAUR63 protein fusions in stable transgenic lines. In addition, SAUR63:YFP:HA protein was mostly peripherally localized in transiently expressing *Nicotiana benthamiana* leaf epidermis cells (S8A Fig).

Western blots using α-HA antibodies against total, soluble, and microsomal (membrane-containing) fractions of protein extracts from whole seedlings revealed that the presumed full-length 48 kD SAUR63:YFP:HA protein was present in both the microsomal and soluble fractions (Figs 3U and S9F). In addition, a protein of about 35 kD was present at roughly equal level as the full-length SAUR63:YFP:HA fusion protein, in the soluble fraction but not in the microsomal fraction. Based on its size and the presence of the HA epitope, this protein likely includes a C-terminal fragment of SAUR63 and the 30 kD YFP:HA tag. Some of the cytoplasmic and nuclear fluorescence seen in confocal images may arise from this soluble truncated protein.

To test whether the N-terminal domain could mediate membrane localization, we made *P*_*35S*_:*SAUR63*^*1-25*^:*YFP*:*HA* plants, expressing fusions of just the N-terminal 25 amino acids of SAUR63 to YFP:HA. In confocal images, the SAUR63^1-25^:YFP:HA fusion protein localized partly to the cell periphery in epidermal cells of roots, cotyledons and tobacco leaves, but with highest fluorescent signal in the nuclei and lower relative intensity at the periphery than seen for the full-length SAUR63:YFP:HA protein (Figs 3G–3J and S7E and S8B). In western blots, SAUR63^1-25^:YFP:HA was found in both microsomal and soluble fractions, but with a higher proportion in the soluble fraction than was seen for full-length SAUR63:YFP:HA (Figs 3U and S9G). These results indicate that the N-terminal 25 amino acid sequence can associate with the PM, but possibly less strongly than does the full-length SAUR63 protein. Moreover, the nuclear localization activity of the N-terminal domain also suggests that additional portions of the full-length protein may normally promote PM over nuclear localization.

To test whether the N-terminal domain is necessary for PM localization, we made *P*_*35S*_:*SAUR63*^*26-142*^:*YFP*:*HA* transgenic plant lines expressing fusion protein that lacked amino acids 2–25 after the N-terminal methionine (SAUR63^26-142^:YFP:HA). These generally had lower full-length fusion protein levels than did *P*_*35S*_:*SAUR63*:*YFP*:*HA* transgenic lines (S9A–S9E Fig), so that high confocal gain settings or prolonged exposure of western blots were usually needed to detect the full-length protein. In confocal images, in root meristem cells SAUR63^26-142^:YFP:HA fluorescence appeared evenly throughout the cytoplasm and nuclei, with no visible concentration near the plasma membrane (Figs 3K–3N, 3S and 3T and S6E–S6H). In cotyledons of stable lines and in transiently expressing tobacco leaf epidermal cells, SAUR63^26-142^:YFP:HA fluorescence appeared partly at the cell periphery (reflecting either PM or cytoplasm) and also in cytoplasmic strands that crossed the vacuole (S7F and S8C Figs). The apparent cytoplasmic localization was much more apparent in transiently expressing tobacco leaf cells than in Arabidopsis cotyledons. In fractionation experiments, SAUR63^26-142^:YFP:HA protein was mainly in the soluble fraction, although a small amount could be detected in microsomal fractions of some blots (Figs 3U and S9G). SAUR63^26-142^:YFP:HA protein also had two smaller proteins (of about 38 and 35 kD) rather than the single major truncated product seen for SAUR63:YFP:HA (Figs 3U and S9F and S9G). These truncated forms appeared in the soluble fraction and were much more abundant than was the full-length protein, and may therefore dominate the fluorescent signal in these plants. Moreover, in the presence of cycloheximide to inhibit new protein synthesis, SAUR63^26-142^:YFP:HA protein disappeared more quickly than did SAUR63:YFP:HA protein, indicating that loss of the N-terminal domain decreases the half-life of the protein (S9H Fig). Together, these results show that the N-terminal domain is required both for accumulation of high levels of full-length SAUR63:YFP:HA fusion protein and for robust PM localization. These properties may be coupled, for example if weaker PM association uncovers residues recognized by the protein turnover machinery, or conversely if a less stable protein is degraded with faster kinetics than it associates with the PM.

### SAUR63 plasma membrane localization promotes growth

To target SAUR63:YFP:HA to the PM independently of its natural N-terminus, we fused the N-terminal 12 amino acids from CBL1 (Calcineurin Binding protein Like 1) to the N-terminally truncated SAUR63^26-142^:YFP:HA protein (Fig 3B). The CBL1^1-12^ sequence is myristoylated and palmitoylated and thereby localizes to the PM [45]. Most *P*_*35S*_:*CBL1*^*1-12*^: *SAUR63*^*26-142*^:*YFP*:*HA* lines had readily detectable fusion protein (S9B and S9E Fig). The full-length CBL1^1-12^:SAUR63^26-142^:YFP:HA protein localized predominantly to the PM in confocal images, and in western blots appeared mostly in the microsomal fraction with weaker signal in the soluble fraction (Figs 3O–3R and 3U and S7H, S8D and S9G). Thus, adding the CBL1^1-12^ N-terminal sequence restored robust membrane association to truncated SAUR63^26-142^:YFP:HA.

**Fig 4:**
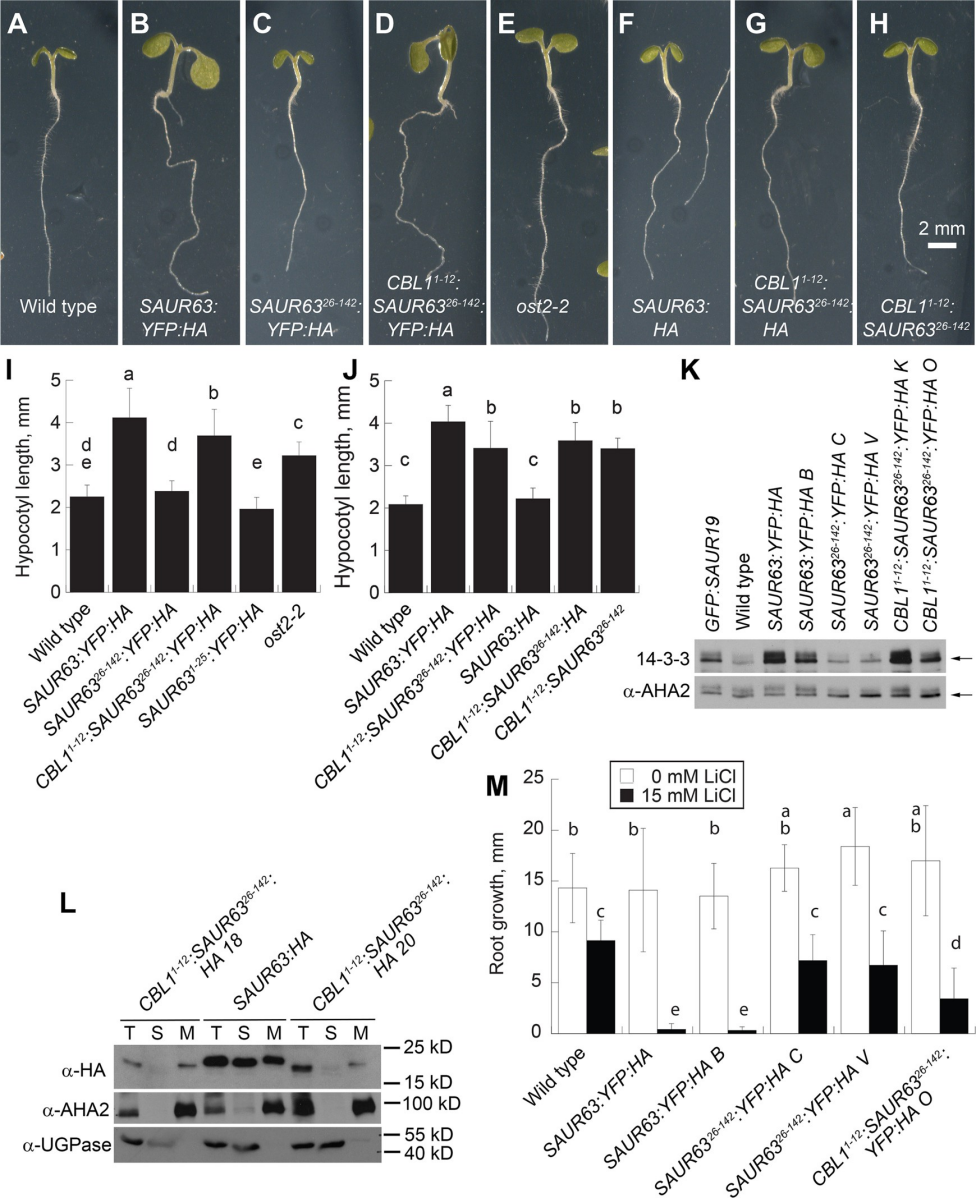
Phenotypic effects of SAUR63 variants with altered N-terminal sequences. **A-H)** Photographs of 5-day-old seedlings of indicated genotypes. Seedlings were grown in long days in presence of sucrose to allow root growth of all genotypes. Scale bar, 2 mm, is shown in panel H and refers to panels A-H. **I,J)** Hypocotyl lengths of seedlings of indicated genotypes grown for 4d in short days. **K)** 14-3-3 overlay western blot of phosphorylated H^+^-ATPase and western blot of overall AHA2 level from total proteins of seedlings of indicated genotypes. Arrows indicate phosphorylated AHA protein (upper blot) or total AHA protein (lower blot). **L)** Fractionation of SAUR63:HA and CBL1^1-12^:SAUR63^26-142^:HA fusion proteins. Two different *P*_*35S*_:*CBL1*^*1-12*^:*SAUR63*^*26-142*^:*HA* lines are shown. T, total protein; S, soluble fraction; M, microsomal fraction; α-HA detects SAUR63 fusion protein; α-AHA2 detects a membrane protein; α-UGPase detects a soluble protein. **M)** Root growth of 5-day-old seedlings grown for 3 days in the absence or presence of 15 mM LiCl. Graphs in I, J, and M show means ± s.d. Letters in graphs indicate statistical classes based on Tukey’s Honestly Significant Difference test. n, from left to right: Panel I: 24, 32, 28, 20, 31, 28; panel J: 23, 22, 24, 26, 19, 25; panel M: 25, 28, 22, 29, 18, 25, 24, 24, 23, 23, 21, 26.

Activity of these SAUR63:YFP:HA fusion protein variants depended on their abundance at the PM. *P*_*35S*_:*CBL1*^*1-12*^: *SAUR63*^*26-142*^:*YFP*:*HA* seedlings had elongated hypocotyls, enlarged cotyledons, tortuous root and dark-grown hypocotyl growth, and short roots in the absence of sucrose, just as did *P*_*35S*_:*SAUR63*:*YFP*:*HA* seedlings (Figs 4A, 4B, 4D, 4I and 4J and S4A, S4B and S4F). In contrast, *P*_*35S*_:*SAUR63*^*26-142*^:*YFP*:*HA* lines expressing N-terminally truncated fusion protein grew similarly to wild-type seedlings (Figs 4C and 4I and S4C and S4F). Moreover, *P*_*35S*_:*CBL1*^*1-12*^: *SAUR63*^*26-142*^:*YFP*:*HA* seedlings, but not *P*_*35S*_:*SAUR63*^*26-142*^:*YFP*:*HA* seedlings, had increased level of Thr^947^-phosphorylated H^+^-ATPase, and were hypersensitive to LiCl (Figs 4K and 4M and S5D). *P*_*35S*_:*CBL1*^*1-12*^: *SAUR63*^*26-142*^:*YFP*:*HA* seedlings also had more acidic root apoplastic pH based on HPTS fluorescence ratio (S5E Fig). These results indicate that membrane association allows SAUR63:YFP:HA protein to promote H^+^-ATPase activity and cell expansion.

The C-terminal YFP:HA tag likely increases SAUR63:YFP:HA fusion protein activity by stabilizing it [15]. To determine whether membrane association alone was sufficient to increase SAUR63 activity, we made plants carrying a *P*_*35S*_:*CBL1*^*1-12*^: *SAUR63*^*26-142*^:*3xHA* transgene with an N-terminal membrane-localizing fatty acylation motif (CBL1^1-12^) and a 39 aa C-terminal epitope tag (3xHA) that likely does not stabilize the protein [15] (Fig 3B). We compared these to plants expressing a *P*_*35S*_:S*AUR63*:*3xHA* transgene having the native N-terminal domain (Fig 3B). *P*_*35S*_:S*AUR63*:*3xHA* seedlings grew similarly to wild-type seedlings, as seen previously for *P*_*SAUR63*_:S*AUR63*:*3xHA* seedlings [15] (Figs 4A, 4F and 4J and S4A and S4B). In contrast, *P*_*35S*_:*CBL1*^*1-12*^: *SAUR63*^*26-142*^:*3xHA* lines had elongated hypocotyls and enlarged cotyledons, similar to albeit weaker than growth phenotypes of *P*_*35S*_:S*AUR63*:*YFP*:*HA* and *P*_*35S*_:*CBL1*^*1-12*^: *SAUR63*^*26-142*^:*YFP*:*HA* lines (Figs 4G and 4J and S4A and S4B). *P*_*35S*_:*CBL1*^*1-12*^: *SAUR63*^*26-142*^:*3xHA* roots grew similarly to those of wild-type plants, but were more sensitive to LiCl (S4B and S5E Figs). In fractionation experiments, the CBL1^1-12^: SAUR63^26-142^:3xHA full-length protein was localized primarily in the microsomal fraction, similarly to CBL1^1-12^:SAUR63^26-142^:YFP:HA; whereas the SAUR63:3xHA full-length protein was present in both soluble and microsomal fractions, similarly to SAUR63:YFP:HA (Fig 4L). In fact, although *P*_*35S*_:*CBL1*^*1-12*^: *SAUR63*^*26-142*^:*3xHA* plants had stronger phenotypes than did *P*_*35S*_: *SAUR63*:*3xHA* plants, the total level of full-length CBL1^1-12^: SAUR63^26-142^:3xHA appeared lower than that of SAUR63:3xHA in the lines assayed (Fig 4L).

Similarly, *P*_*35S*_:*CBL1*^*1-12*^: *SAUR63*^*26-142*^ lines overexpressing a membrane-targeted fusion protein without any C-terminal epitope tag had elongated hypocotyls and enlarged cotyledons (Figs 4H and 4J and S4A, S4D and S4E). *P*_*35S*_:S*AUR63* lines had weaker phenotypes than those of *P*_*35S*_:*CBL1*^*1-12*^: *SAUR63*^*26-142*^ lines, with slightly elongated hypocotyls and enlarged cotyledons (S4D and S4E Fig). While we could not measure levels of SAUR63 or CBL1^1-12^: SAUR63^26-142^ proteins lacking a C-terminal epitope tag, these results suggest that forced PM localization, achieved by replacing the natural N-terminal domain with the CBL1^1-12^ N-terminal fatty acylation motif, increased the potency of SAUR63, as it did for SAUR63:3xHA. The similar phenotypes of *P*_*35S*_:*CBL1*^*1-12*^: *SAUR63*^*26-142*^ and *P*_*35S*_:*CBL1*^*1-12*^: *SAUR63*^*26-142*^:*3xHA* lines suggest that the 3xHA C-terminal tag did not have a large influence on protein function.

### The SAUR63 N-terminal domain interacts with plasma membrane anionic phospholipids

**Fig 5:**
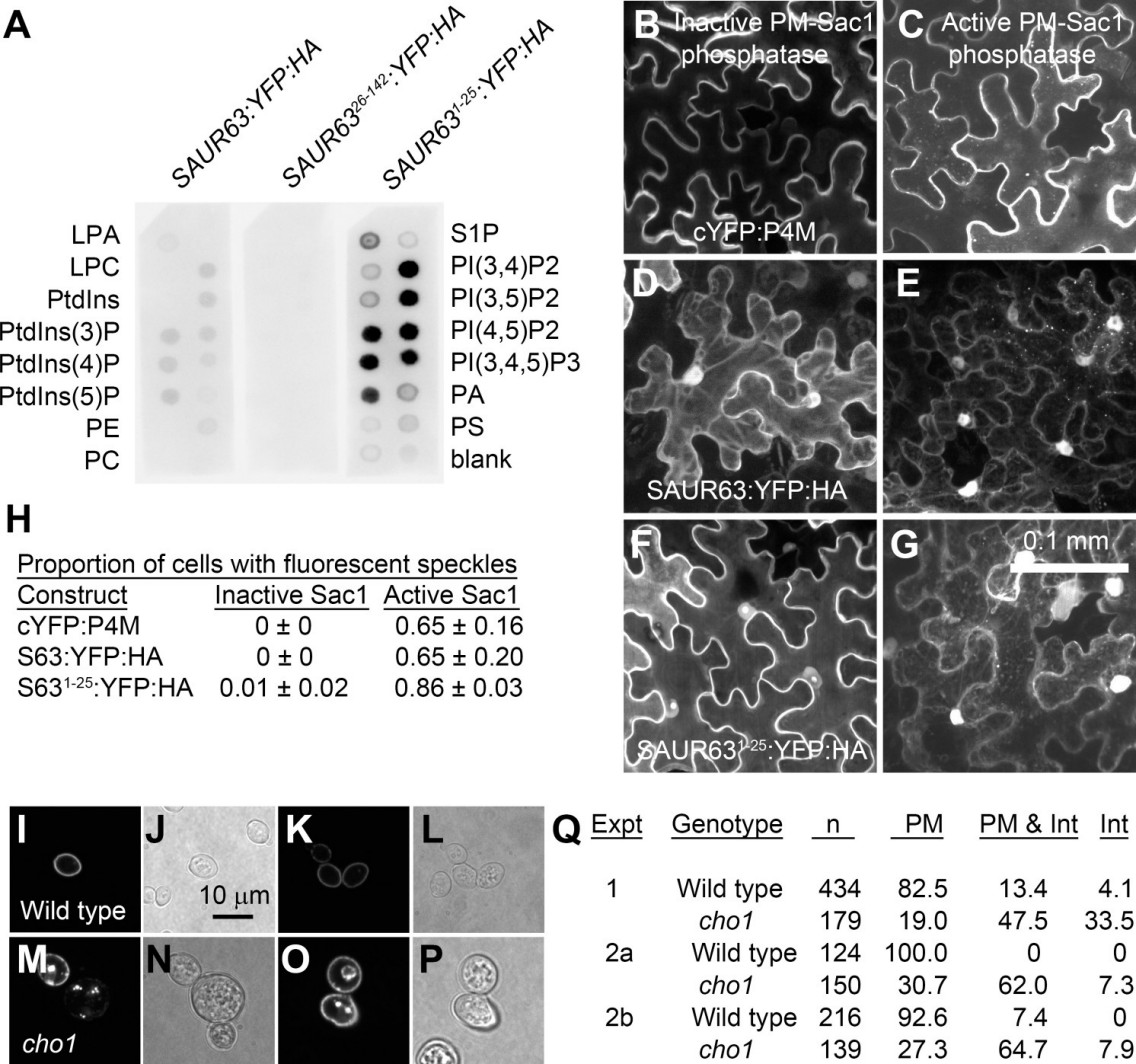
Effects of changes in anionic phospholipids on SAUR63 localization. **A)** Lipid blots exposed to proteins extracted from SAUR63:YFP:HA, SAUR63^26-142^:YFP:HA, or SAUR63^1-25^:YFP:HA plants and then probed with α-HA antibody. S10B Fig shows a longer exposure of the SAUR63:YFP:HA and SAUR63^26-142^:YFP:HA strips. An independent experiment using only SAUR63:YFP:HA and SAUR63^26-142^:YFP:HA gave similar results. **B-G)** Representative images showing localization of indicated fusion proteins in transiently expressing *Nicotiana benthamiana* leaves coexpressing active or inactive mutant PM-localized yeast PI(4)P phosphatase MAP-mCherry:Sac1. cYFP:P4M is a control indicator protein that binds PI(4)P. Shown are confocal image Z-stack projections that span the depth of the targeted epidermal cell. Scale bar, 0.1 mm. **H)** Proportion of *Nicotiana benthamiana* leaf cells expressing indicated fusion proteins and inactive or active MAP-mCherry:Sac1 having visible fluorescent intracellular speckles. Data are means ± s.d. of 3 replicate experiments. Each data point represents 28–70 cells that were doubly transfected as indicated by presence of both yellow (SAUR63:YFP:HA) and red (MAP-mCherry:Sac1) signals. **I-Q)** Localization of SAUR63:eGFP in wild-type and *cho* mutant yeast. **I-P)** Representative paired fluorescent (eGFP) or bright-field images of wild type (I-L) or *cho1* mutant (M-P) yeast, showing examples of eGFP localization in PM only (I,K), both in PM and intracellular (M upper left cell, O both cells), or only intracellular (M, cell in the center of the image). Scale bar, 10 μm. **Q)** Table showing percent of cells in each category for three experiments. Experiments 2a and 2b were from the same yeast transformation, data gathered from different transformed colonies on different days. Int, intracellular.

Protein domains that are rich in basic and hydrophobic amino acids can associate with anionic membrane phospholipids through electrostatic interactions [35,41,43]. Such domains may be unstructured, or may form stable or induced amphipathic α-helices with basic residues on one face that contact anionic phospholipid head groups. To assess whether SAUR63:YFP:HA and its N-terminal domain could bind to phospholipids, we probed lipid blots with extracts from plants expressing these fusion proteins. Extracts from *P*_*35S*_:*SAUR63*:*YFP*:*HA* and *P*_*35S*_:*SAUR63*^*1-25*^:*YFP*:*HA* plants bound to several anionic lipids on the blot, including phosphatidylserine and all of the phosphatidylinositol phosphates (Fig 5A). Weak or no binding was detected to phosphatidic acid (PA), phosphatidylethanolamine (PE), phosphatidylcholine (PC), or phosphatidylinositol (PtdIns). Extracts from *SAUR63*^*26-142*^:*YFP*:*HA* plants had no detectable binding. While the lower level and apparent instability of SAUR63^26-142^:YFP:HA protein in vivo (S9H and S10A Figs) may have reduced signal in this experiment, even prolonged exposure of the blots with *P*_*35S*_:*SAUR63*^*26-142*^:*YFP*:*HA* extracts failed to yield appreciable signal (S10B Fig). Moreover, SAUR63:YFP:HA and SAUR63^26-142^:YFP:HA proteins each appeared stable for at least 1h in the buffer used to make the protein extracts for the lipid blot assays (S10C Fig). These results suggest that the N-terminal domain of SAUR63 can bind to anionic phospholipids, possibly directly.

**Fig 6:**
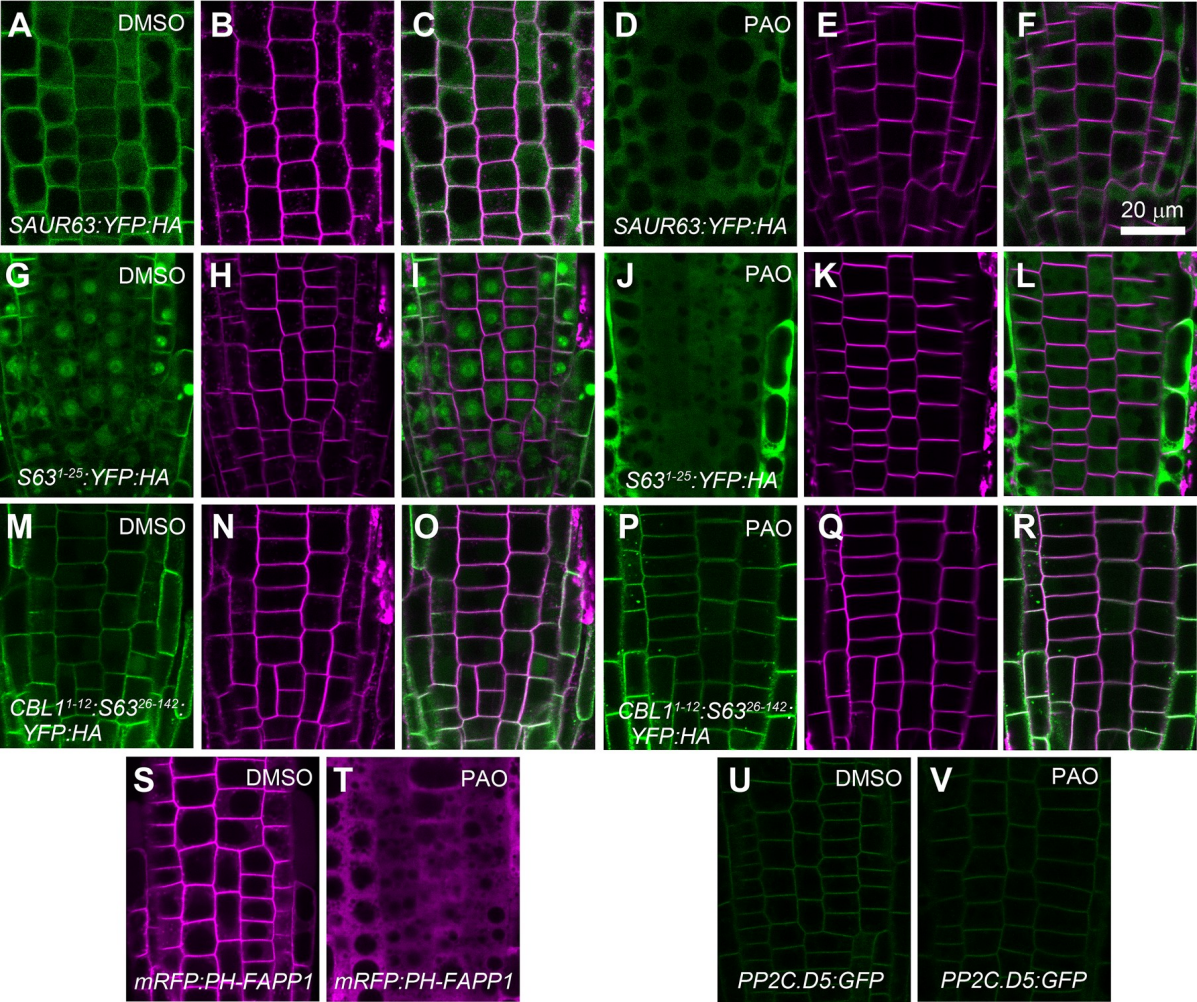
Effect of PAO treatment on plasma membrane localization of SAUR63:YFP fusion proteins. **A-F)** *P*_*35S*_:*SAUR63*:*YFP*:*HA*. **G-L)** *P*_*35S*_:*SAUR63*^*1-25*^:*YFP*:*HA*. **M-R)** *P*_*35S*_:*CBL1*^*1-12*^:*SAUR63*^*26-142*^:*YFP*:*HA*. **S,T)** *P*_*35S*_:*mRFP*:*PH-FAPP1*. **U,V)** *P*_*PP2C*.*D5*_:*PP2C*.*D5*:*GFP*. Roots were treated with DMSO (A-C,G-I,M-O,S,U) or the PI4K inhibitor PAO (D-F,J-L,P-R,T,V) for 30 minutes before imaging root meristem cells. Shown are YFP fluorescence (green, A,D,G,J,M,P), FM4-64 staining red fluorescence (magenta, B,E,H,K,N,Q), and both YFP and FM4-64 signals together (C,F,I,L,O,R). In S and T, magenta fluorescence shows the mRFP:PH-FAPP1 control PI(4)P-binding fusion protein. In U and V, green fluorescence shows the PP2C.D5:GFP protein. Scale bar, 20 μm.

To test the importance of anionic phospholipids for localization of SAUR63 having an intact N-terminal domain, we manipulated membrane phospholipid composition in several contexts and then observed effects on SAUR63:XFP localization. The primary phosphoinositide in plant plasma membranes is PI(4)P, which is produced mainly by phosphorylation of PI by phosphatidylinositol-4-kinase (PI4K) [46,47]. Treatment with the putative PI4K inhibitor phenylarsine oxide (PAO) caused release from PM to cytoplasm of the PI(4)P indicator mCherry:PH-FAPP1 [48,49], full-length SAUR63:YFP:HA, and SAUR63^1-25^:YFP:HA in root meristem epidermal cells; but did not displace CBL1^1-12^: SAUR63^26-142^:YFP:HA (Fig 6A–6T). PAO also failed to displace PP2C.D5:GFP protein from the PM (Fig 6U and 6V), suggesting that PP2C.D5 associates with the PM independently of PI(4)P lipid content.

As mentioned above, transiently expressed SAUR63:YFP:HA and SAUR63^1-25^:YFP:HA fusion proteins appeared mainly at the cell periphery in *Nicotiana benthamiana* leaves (S8A and S8B Fig). To decrease PI(4)P at the PM in these cells, we coexpressed MAP-mCherry:Sac1, which has the catalytic domain of the yeast *Saccharomyces cerevisiae* PI(4)P phosphatase targeted to the PM by fusion with a myristoylation and palmitoylation (MAP) motif. Under this condition, SAUR63:YFP:HA and SAUR63^1-25^:YFP:HA associated partially with internal speckles, as did the PI(4)P indicator cYFP:P4M [46,50] (Fig 5C, 5E, 5G and 5H). These results suggest decreased association with the PM and putative binding instead to anionic phospholipids on intracellular compartments that are also electrostatic [46,47]. In control assays in cells expressing a catalytically inactive mutant version of MAP-mCherry:Sac1, cYFP:P4M, SAUR63:YFP:HA and SAUR63^1-25^:YFP:HA fluorescence were present at the cell periphery, but were not associated with speckles (Figs 5B, 5D, 5F and 5H). Using either active or inactive MAP-mCherry:Sac1, SAUR63:YFP:HA and SAUR63^1-25^:YFP:HA fluorescence was also seen in the nucleus and in cytoplasmic strands, suggesting presence of a pool of soluble SAUR63:YFP:HA or its breakdown products, as also seen in the stable lines.

Finally, SAUR63:eGFP fusion protein expressed in yeast *S*. *cerevisiae* also associated with the plasma membrane (Fig 5I–5L and 5Q). This result indicates that SAUR63 can bind to membranes in the absence of other plant proteins. In a *cho1* mutant with decreased levels of phosphatidylserine (PS), the major anionic lipid in the yeast PM, the SAUR63:eGFP fusion protein in addition localized partly or entirely to intracellular structures (Fig 5M–5Q)[46,51].

These results indicate that SAUR63:XFP interacts with anionic phospholipids in the plasma membrane, including PI(4)P in plant root or shoot epidermal cells, or PS when expressed in yeast. As the fusion proteins can bind to both PI(4)P and PS, the interaction may depend on electrostatic interactions rather than specific binding to particular lipid head groups.

### SAUR domain mutations affect intracellular localization

**Fig 7:**
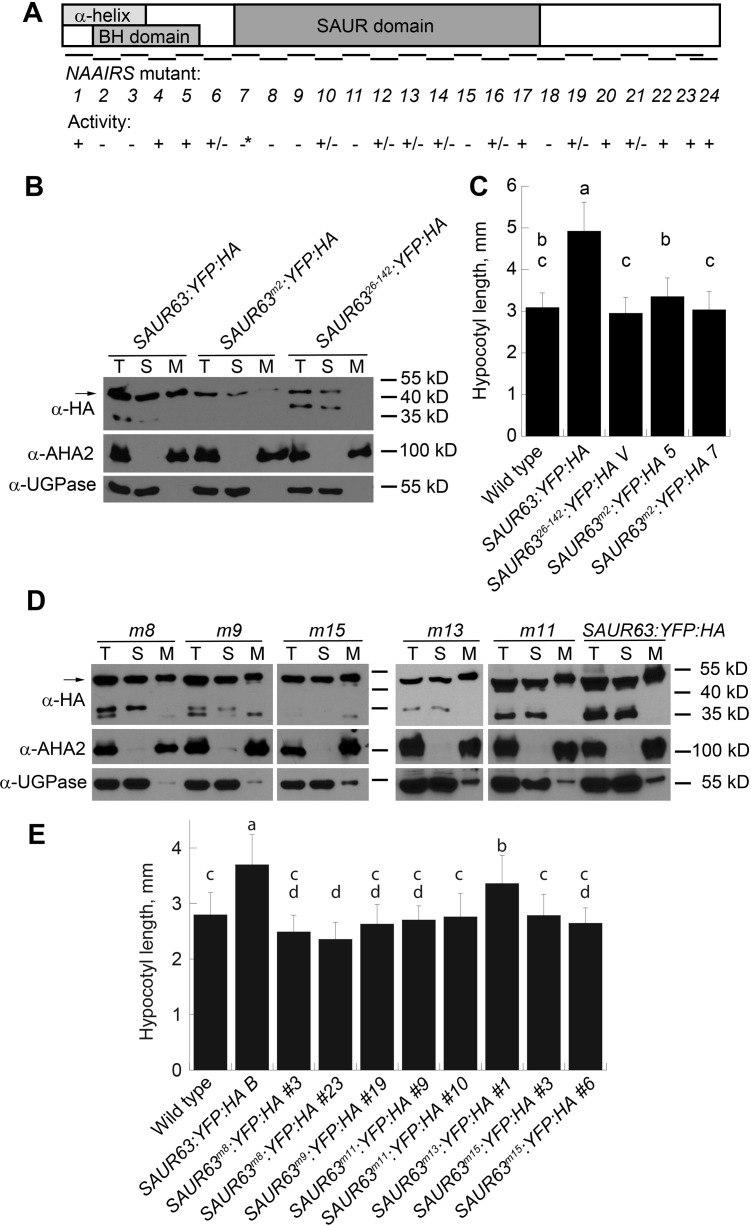
Effects of mutations on SAUR63 properties. **A)** Diagram of SAUR63 indicating predicted structural features, locations of NAAIRS mutations *m1*-*m24*, and whether SAUR63:CerFP:HA and/or SAUR63YFP:HA fusion proteins with each mutation retained function, based on their ability to confer either tortuous root growth or increased hypocotyl elongation in Figs 7C and 7E and S11 and S1 Table. +, caused phenotypes in all experiments; -, failed to cause phenotypes in all experiments; +/- caused phenotypes in at least one experiment; *, no fusion protein detected. **B,D)** Fusion proteins in stable *P*_*35S*_:*SAUR63*^*NAAIRS*^:*YFP*:*HA* lines, detected by western blots in total (T), soluble (S) and microsomal (M) protein fractions. α-HA detects SAUR63:YFP:HA fusion protein variants; α-AHA2 detects a membrane protein; α-UGPase detects soluble proteins. In D, *P*_*35S*_:*SAUR63*^*NAAIRS*^:*YFP*:*HA* line names are abbreviated by the mutation name (*m8* etc.). **C,E)** Hypocotyl lengths of stable lines of seedlings grown for 4d in short days. Graph shows means ± s.d. Letters indicate statistical classes based on Tukey’s Honestly Significant Difference test. n, from left to right: panel C: 55, 30, 34, 31, 36; panel E: 17, 24, 29, 21, 24, 27, 29, 24, 29, 30.

To identify additional structural determinants needed for SAUR63 function, we substituted blocks of 6 amino acids with the amino acid sequence NAAIRS (historically considered to be compatible with multiple different protein folds) [52,53]. 24 mutations called *m1*-*m24* were constructed to cover the length of SAUR63, starting with amino acid 2 after the N-terminal methionine, in the context of a *P*_*EST*_:*SAUR63*:*CerFP*:*HA* estradiol-inducible expression construct [22](S2 Table and Fig 7A). *P*_*EST*_:*SAUR63*:*CerFP*:*HA* seedlings expressing the wild-type fusion protein had tortuous root growth on plates containing estradiol, similarly to *P*_*35*_:*SAUR63*:*YFP*:*HA* plants, although they did not have clearly increased hypocotyl or cotyledon growth, possibly due to poor induction of the transgene in shoot tissues (S11A Fig). Whereas most of the *NAAIRS* mutant fusions increased root tortuosity as did the wild-type fusion protein construct, plants expressing the mutant constructs *m2*, *m3*, *m8*, *m9*, *m11*, *m13*, *m15* and *m18* consistently lacked tortuous root growth despite expressing detectable levels of the fusion protein (Figs 7A and S11A and S11B and S2 Table). The expected size fusion protein was not detectable in plants with constructs carrying mutation *m7*, which was therefore not informative regarding structural requirements for function.

**Fig 8:**
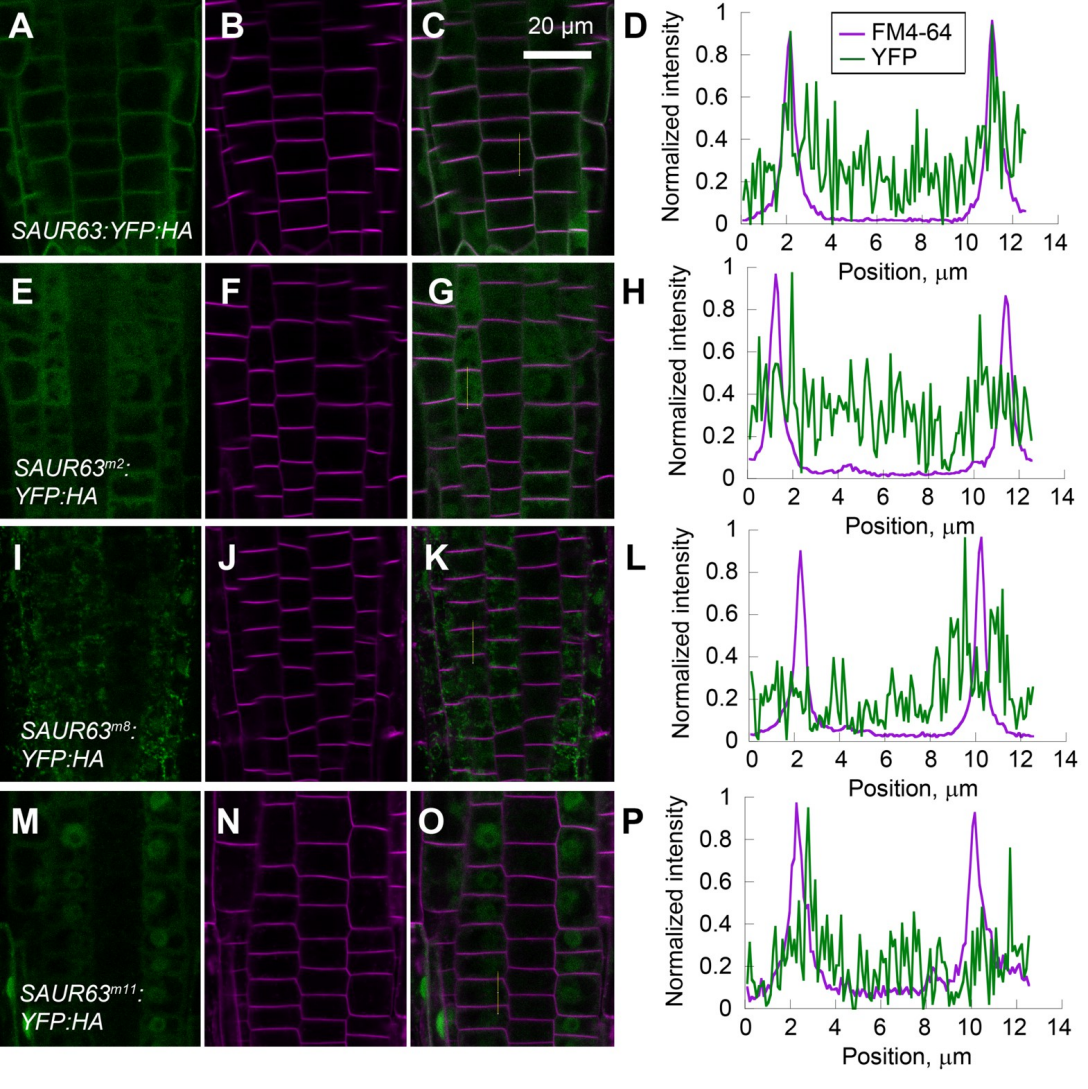
Effects of *NAAIRS* mutations on SAUR63:YFP:HA localization in root meristem cells. **A-D)** *P*_*35S*_:*SAUR63*:*YFP*:*HA*. **E-H)** *P*_*35S*_:***S****AUR63*^*m2*^:*YFP*:*HA*. **I-L)** *P*_*35S*_:*SAUR63*^*m8*^:*YFP*:*HA*. **M-P)** *P*_*35S*_:SAUR63^m11^:YFP:HA. Shown are fluorescence confocal microscopy images of YFP (green, A,E,I,M), FM4-64 membrane staining (magenta, B,F,J,N), and both channels together (C,G,K,O) with vertical yellow lines indicating locations of quantitation of fluorescence intensity signals, scaled to the maximum signal along the line (D,H,L,P). Image color channel brightnesses were adjusted for visibility. S12 Fig shows images for additional genotypes analyzed in the same experiment. Scale bar, 20 μm.

The *m2* mutation and to a lesser extent the *m3* mutation are predicted to decrease both the basic hydrophobic score of the N-terminal domain and the predicted amphipathic α-helix hydrophobic moment, without preventing α-helix formation (S1 Table). To test how the *m2* mutation affects localization, we made *P*_*35S*_:*SAUR63*^*m2*^:*YFP*:*HA* Arabidopsis plant lines, and also transiently transformed tobacco leaves with this construct. Similarly to SAUR63^26-142^:YFP:HA, SAUR63^m2^:YFP:HA protein localized to the cytoplasm in root meristem cells, and to the periphery and cytoplasmic strands in cotyledon and tobacco leaf cells (Figs 8E–8H and S7G, S8E and S12E–S12H). Also similarly to SAUR63^26-142^:YFP:HA, in western blots, SAUR63^m2^:YFP:HA protein had low abundance and appeared mainly in the soluble fraction (Fig 7B). Moreover, *P*_*35S*_:*SAUR63*^*m2*^:*YFP*:*HA* lines had hypocotyl lengths indistinguishable from those of wild-type plants (Fig 7C). These results support the model that an N-terminal amphipathic α-helix promotes PM localization and is required for growth-promoting activity.

Most other mutations that affected activity in this survey fell in the conserved SAUR domain (Fig 7A). To test the importance of this domain, we introduced mutations *m8*, *m9*, *m11*, *m13*, and *m15* into the *P*_*35S*_:*SAUR63*:*YFP*:*HA* context, and established plant lines expressing these (Fig 7D). Lines expressing *P*_*35S*_:*SAUR63*:*YFP*:*HA* with mutations *m8*, *m9*, *m11*, or *m15* resembled wild-type plants, whereas *P*_*35S*_:*SAUR63*^*m13*^:*YFP*:*HA* plants had more elongated hypocotyls as *P*_*35S*_:*SAUR63*:*YFP*:*HA* plants did (Fig 7E). Thus, the *m13* mutant protein retained activity (despite initial results with *P*_*EST*_:*SAUR63*^*m13*^:*CerFP*:*HA* plants), whereas the *m8*, *m9*, *m11*, and *m15* mutations decreased growth-promoting activity.

We explored effects of these SAUR domain mutations on intracellular localization. SAUR63:YFP:HA fusion proteins with *m8*, *m9*, *m11*, *m13*, and *m15* mutations fractionated partially in the microsome fraction, similarly to wild-type fusion protein (Fig 7D). However, by confocal microscopy, fusion proteins carrying these mutations sometimes had lower PM association than did the wild-type SAUR63:YFP:HA, depending on which cells were assayed. In root meristem cells, only mutant protein m13 had a small fraction of apparent PM localization, although it appeared mainly cytoplasmic; mutant proteins m8 and m9 appeared predominantly as intracellular speckles, possibly reflecting association with organelles; and mutant proteins m11 and m15 appeared diffusely in the cytoplasm and nuclei (Figs 8I–8P and S12I–S12T). Thus, in root cells, the SAUR domain mutations reduced PM localization.

In cotyledons, m8 and m9 mutant proteins appeared weakly around the cell periphery, and in brighter speckles nearby; m11 and m15 mutant proteins appeared at the cell periphery, and in some cytoplasmic strands; and m13 protein appeared at the cell periphery (S7I–S7M Fig). While the peripheral fluorescent signals in cotyledons are consistent with PM localization, visible fluorescence in speckles and/or cytoplasmic strands suggests significant cytoplasmic localization of the mutant fusion proteins and/or their breakdown products. In transient assays in tobacco leaves, each of these mutant proteins appeared mostly at the cell periphery and in nuclei, but with little visible cytoplasmic localization, in contrast to the m2 mutant fusion protein (S8E, S8F, S8G, S8I, S8J and S8K Fig). We also used transient assays to test localization of double mutant fusion proteins lacking N-terminal amino acids 2–25 and also mutated in the SAUR domain. Both SAUR63^26-142/m9^:YFP:HA and SAUR63^26-142/m15^:YFP:HA proteins localized predominantly in the cytoplasm in *N*. *benthamiana* cells (S8H and S8L Fig), similarly to SAUR63^26-142^:YFP:HA or SAUR63^m2^:YFP:HA, but in contrast to the SAUR63^m9^:YFP:HA and SAUR63^m15^:YFP:HA variants with mutations in the SAUR domain only. Thus, in *N*. *benthamiana* leaf cells, the N-terminal domain apparently still mediated PM association in the absence of an intact SAUR domain. Overall, the results suggest that SAUR63 may localize to the PM by slightly different manners in different tissues. In root meristem cells, the SAUR domain and the N-terminal domain each contribute to PM localization; whereas in *N*. *benthamiana* leaf cells, the N-terminal domain may be more essential.

## Discussion

Our results show that SAUR63 must be present at the PM to stimulate cell expansion. Mutation or deletion of the N-terminal domain decreased both PM association and growth-promoting activity. Conversely, replacing the native N-terminal domain of SAUR63 with the CBL1^1-12^ fatty-acylated sequence caused persistent PM localization and restored growth-promoting activity to N-terminally truncated SAUR63^26-142^:YFP:HA. PM targeting by CBL1^1-12^ also increased activity of SAUR63^26-142^ or SAUR63^26-142^:3xHA proteins lacking a stabilizing C-terminal YFP fusion. Thus either forced PM localization or C-terminal: XFP fusion, which increases the half-life of the protein [15], can increase SAUR63 activity.

SAUR63 localizes to the PM in part through interactions between the N-terminal domain and phospholipids. The first 25 amino acids of SAUR63 containing a predicted amphipathic α-helix could associate with the PM, and could bind to anionic phospholipids that mark the PM including PI(4)P and PS [46,47]. Perturbing PM anionic lipid pools in plant or yeast cells displaced SAUR63 fusion proteins to the cytoplasm or to intracellular speckles. That SAUR63 associates with the PM both in plant and in yeast cells suggests that the interaction does not depend on other plant proteins, and occurs through electrostatic attraction. Beyond the SAUR63 clade, several other SAUR proteins have N-terminal domains with predicted BH domains and amphipathic α-helices, and may similarly localize in part through protein-lipid interactions. The diversity of N- and C-terminal domain characteristics among different SAUR proteins may confer distinct localization and regulatory properties, and it will be interesting to learn how SAUR proteins from other clades localize.

**Fig 9:**
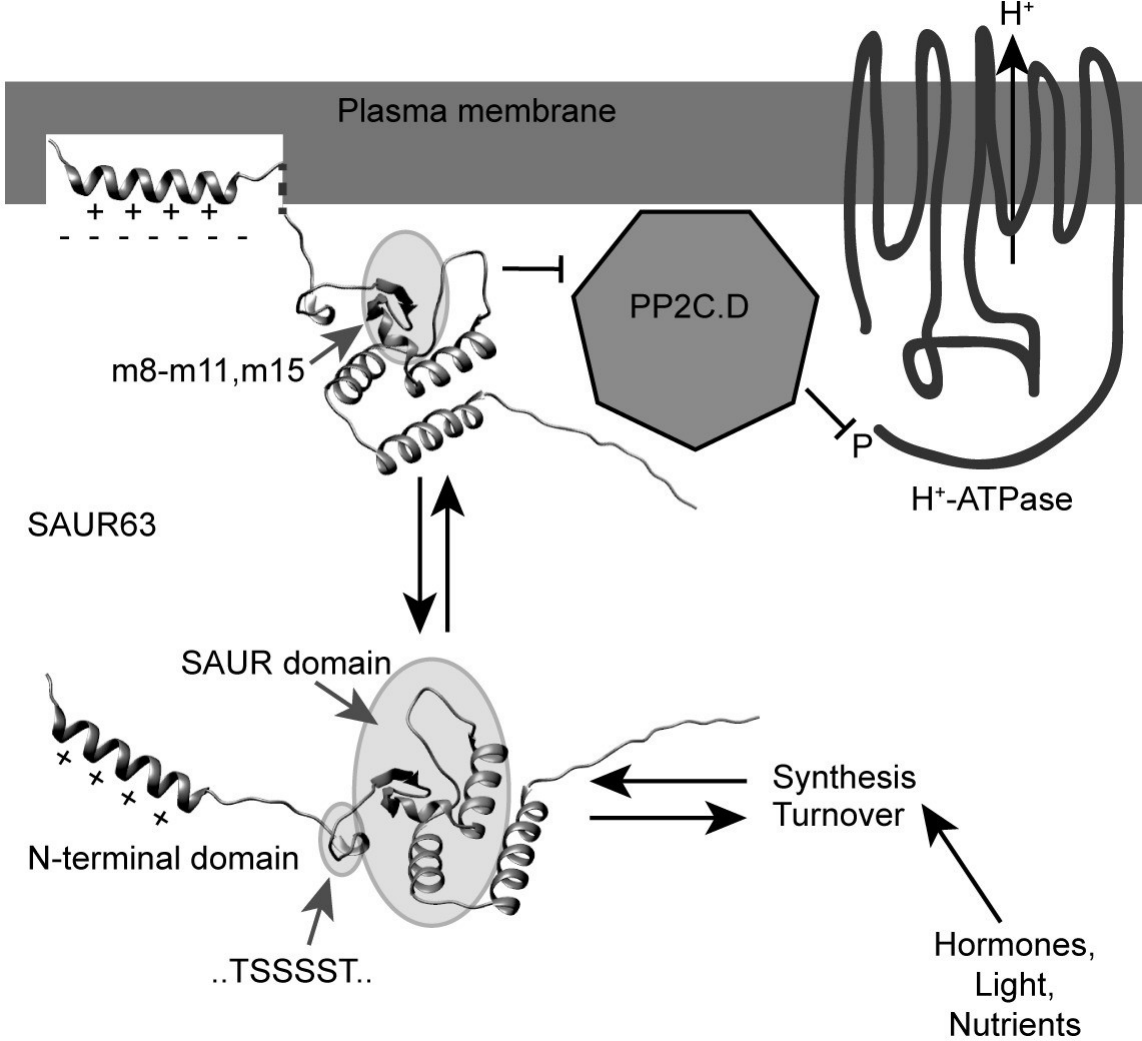
Model for SAUR63 dynamics. At lower center is a computationally predicted structure for SAUR63 with the N-terminal domain indicated and the SAUR domain surrounded by a shaded oval. The protein likely partitions dynamically between the cytosol (lower image) and PM association (upper image). The predicted basic amphipathic α-helix at the N-terminus (+ signs indicate positively charged face) can interact with the PM through electrostatic attraction to anionic phospholipids (- signs), and likely embedding of the hydrophobic face within the lipid bilayer. In addition, the conserved SAUR domain may interact with the PM through its association with PP2C.D or other PM-associated proteins. In the upper diagram, the shaded oval indicates the locations of amino acids corresponding to *NAAIRS* mutations *m8*-*m11* and *m15* in the predicted structure, which include sequences that affect both PM localization and growth-promoting activity. Orientations of the N-terminal and SAUR domains were adjusted independently in the upper diagram to illustrate these putative interactions with the PM. The linker between the N-terminal domain α-helix and the SAUR domain may be flexible enough to allow these orientations. This linker also has a stretch of six consecutive phosphorylatable amino acids (TSSSST, location indicated by small shaded oval in the lower diagram). Inhibition of PP2C.D proteins by SAUR63 would prevent dephosphorylation of the PM H^+^-ATPase, thereby maintaining its activity for acid growth (26). Rapid partitioning between PM-associated and soluble pools, as well as fast synthesis and turnover of SAUR63 and other SAURs, would allow sensitive regulation of activity by changes in signaling by hormones, light, or nutrients. The computational model of SAUR63 structure was taken from the Alphafold 2.0 website [[39,81], https://alphafold.ebi.ac.uk/entry/F4I1H5] and visualized and oriented using Chimera software [82], https://www.rbvi.ucsf.edu/chimera].

Although the SAUR63^1-25^:YFP:HA N-terminal domain fusion protein associated with the PM, it appeared to do so less strongly than did full-length SAUR63:YFP:HA protein, suggesting that other portions of SAUR63 likely also contribute to PM localization. Several variant proteins with mutations in the SAUR domain localized weakly to the PM in some cells despite having an intact N-terminal domain. In particular, in root meristem cells, *m8* and *m9* mutant proteins localized to internal speckles, whereas *m11* and *m15* mutant proteins localized broadly throughout the cell. It is possible that mutations in the SAUR domain affect the N-terminal domain conformation or accessibility for lipid binding, perhaps at the same time decreasing its nuclear localization activity. The *m8* and *m9* mutations might further create or expose protein surfaces that cause aggregation or that have affinity for intracellular organelles. However, analyses of SAUR63^26-142/m9^:YFP:HA and SAUR63^26-142/m15^:YFP:HA proteins suggested that in *N*. *benthamiana* leaves the N-terminal domain apparently still contributed to PM localization of m9 and m15 SAUR domain mutant fusion proteins; and in fractionation studies from Arabidopsis seedling extracts the SAUR domain mutant proteins still had a substantial proportion in the microsomal fraction. An alternative possible explanation is therefore that the SAUR domain binds to PP2C.D or other membrane-associated proteins while the N-terminal domain binds to anionic lipids, and together these mediate robust PM localization (Fig 9). Consistent with this model, other workers have found that the SAUR domains of SAUR17 and SAUR50 interacted with PP2C.D1 in yeast two-hybrid assays [12]. In this model, the coincidence of both binding activities would concentrate SAUR63 at the PM where it can then engage its regulatory targets. Apparently lower PM association of SAUR domain mutants in root than in leaf cells might reflect differing anionic lipid composition or target protein concentrations at the PM. Similar coincidence mechanisms bring signaling partners together in many membrane-associated signaling pathways. The SAUR domain might then inhibit PP2C.D5 and other PM-associated PP2C.D phosphatases to increase or maintain H^+^-ATPase activity, and may also have additional targets that remain to be discovered [12,19].

In addition to abrogating PM association, removing the N-terminal 25 amino acids substantially decreased levels of SAUR63:YFP:HA fusion protein, likely by increasing protein turnover. SAUR63^26-142^:YFP:HA had a shorter half-life than did SAUR63:YFP:HA, as well as a higher proportion of putative breakdown products in extracts. The N-terminal domain might stabilize the protein by protecting residues that are vulnerable to ubiquitylation and/or proteolytic cleavage, or PM association may sequester SAUR63 from the turnover machinery. That the CBL1^1-12^ sequence increased the fraction of fusion protein that was PM-associated and also decreased the proportion of visible putative breakdown products suggests that PM association may stabilize the protein, and thus that the soluble pool may be turned over more quickly than the PM-associated pool.

Wild-type SAUR63 likely turns over with a half-life of about 10 minutes [15], and should exchange dynamically between soluble and PM-associated pools. The transience of SAUR63 membrane association and its short half-life would permit fast regulation of SAUR63 and hence H^+^-ATPase activity. For example, physiological changes in PM lipid content [54–58], or binding of N-terminal amphipathic α-helix sequences to Ca^2+^/calmodulin [59,60], might regulate SAUR protein PM localization or abundance. Ambient light conditions can also affect SAUR63 turnover rate [15]. Also of note, amino acids 35–40 of SAUR63 comprise a stretch of 6 consecutive serines and threonines that if phosphorylated might affect localization or stability. Consistent with this possibility, NAAIRS mutation *m7* changes amino acids 38, 39, and 40, and appeared to decrease protein stability.

Multiple organs of *P*_*35S*_:*SAUR63*:*YFP*:*HA* and *P*_*35S*_:*CBL1*^*1-12*^:*SAUR63*:*YFP*:*HA* plants grew excessively, including hypocotyls and cotyledons (in presence of sucrose), and their hypocotyls and roots also grew tortuously. These growth phenotypes presumably arise because increased SAUR63 overcomes or bypasses normal homeostatic regulatory mechanisms and increases sink strength. In wild-type plants, auxin induces growth-associated *SAUR* genes within minutes, and many growth-associated *SAUR* transcripts and SAUR proteins (where tested) often also have half-lives of a few minutes [15,16,61–64]. Similarly, many *SAUR* genes are induced quickly in hypocotyls or cotyledons in response to light conditions that regulate growth allocation [5,7]. Moreover, sucrose can regulate several hormone transport or response pathways that can affect *SAUR* expression [65–67]. Thus, in wild-type plants, diverse hormonal, nutritional, and exogenous signals may integrate to determine *SAUR* expression levels and hence the extent of organ growth. In addition, auxin and other signals regulate kinases that phosphorylate PM H^+^-ATPases [26,27,68]. SAUR activity must interact with these pathways to ensure balanced growth that is tuned to environmental conditions and available energy supply. Further work with loss-of-function *saur* mutants may help to elucidate the physiological roles of the many *SAUR* genes in these regulatory networks.

## Materials and methods

### Cloning and generation of transgenic lines

All Arabidopsis lines were in the Columbia (Col-O) ecotype. Targeted *saur* mutants were made by two rounds of CRISPR/Cas9-targeted mutagenesis using sgRNAs with guide sequences i) GCTCAACCGCTGCAGAGAA, to make a 4.5 kb deletion between *SAUR61* and *SAUR64*; and ii) AACTCTTCTTCAGATATCT, to generate mutations in the remaining genes of the clade (S1A and S1F Fig). Guide RNAs were cloned into pDONR207 vector (Invitrogen) using primers listed in S3 Table, and subcloned into binary vector pCUT6 [69] by LR clonase (Invitrogen). For fusion proteins, entry clones were constructed using Gateway Technology in pENTR D-TOPO vector (Invitrogen 45–0218) using primers listed in S3 Table. NAAIRS (Asn Ala Ala Ile Arg Ser) mutant entry clones were generated by site-directed PCR mutagenesis of a *SAUR63* pENTR clone using primers listed in S3 Table. Expression clones listed in S4 Table were constructed by recombination between an entry clone and a destination vector using Gateway LR Clonase II enzyme mix (Invitrogen 11791–020). Constructs were introduced into *Arabidopsis thaliana* ecotype Columbia plants using *Agrobacterium tumefaciens* strain GV3101 pMP90 by floral dip [70]. T1 transformed plants were selected on soil by spraying with 0.011% Finale Herbicide (Glufosinate ammonium 11.33%, Bayer Environmental Science, RTP, NC, product no. 4193473), or selected on agar plates containing 1x MS salts, 1% Sucrose with either 50 μg/ml Kanamycin or 0.00113% Finale. For CRISPR/Cas9-targeted genomic mutations, mutated plants were identified in the T2 generation by PCR-based screening for size polymophisms and by sequencing PCR products spanning the targeted sites, using primers listed in S3 Table. For *P*_*35S*_:*SAUR63*:*x* fusion protein lines, between 15 and 54 lines were generated for each construct, and lines were selected that segregated 3:1 for herbicide or antibiotic resistance and had detectable protein expression by fluorescence microscopy and/or western blot. Different lines are given letter or number designations to distinguish them. Where not otherwise designated, the “FL” *P*_*35S*_:*SAUR63*:*YFP*:*HA* reference line shown in S9 Fig was used. Microscopy images shown are from the same lines as used for the physiology experiments presented.

### Plant growth conditions and phenotypic analyses

Seeds were surface-sterilized with 95% ethanol followed by bleach solution (2:1 H_2_O:bleach with two or three drops of Tween-20), plated on Murashige and Skoog (MS) salts (Murashige and Skoog, 1962) containing 1% (w/v) sucrose and 0.6% (w/v) Phyto-agar (pH 5.7), cold stratified for 1 to 3 days at 4°C, then grown at 22°C with 16h:8h light:dark (long day) or 8h:16h (short day) photoperiod. For hypocotyl length measurements, seedlings were instead grown on vertically oriented plates containing 0.5x MS salts 0.6% Phyto-agar (pH 5.7). For cotyledon area measurements, cotyledons were excised and flattened on double-sided sticky tape before imaging. Hypocotyl, cotyledon, and root growth experiments were performed at least two times for each genotype with similar results. Hypocotyl or root tortuosity index was calculated as (contour length—distance between ends)/(contour length), equivalent to 1-distance/length [15]. This tortuosity index differs from previous measures of tortuosity [71,72] but is convenient to calculate and to graph because it varies from 0 to 1. To minimize possible effects of fractal degree in the contour length measurements, the same person measured each seedling at the same magnification within each experiment. For LiCl sensitivity experiments, seedlings were first grown for 3 or 5 days on vertically oriented MS 1% Sucrose plates, then transferred to plates containing 15 mM LiCl (also containing 10 μM estradiol, for the *P*_*EST*_:*SAUR63*:*CerFP*:*HA* line), and root growth over the following three days was measured. Images were obtained using a flatbed scanner or using a Leica DFC420 camera on a Wild dissecting microscope, and organ dimensions were measured using Fiji (Image J) software [73]. To measure hypocotyl epidermal cell dimensions, plant lines expressing the shoot epidermal PM marker *P*_*ML1*_:*RFP* [74] were grown on vertically oriented plates, and z-stack confocal image projections were taken on a Leica sp8 confocal microscope as described previously [75].

### Transient assays in Nicotiana benthamiana

*Agrobacterium tumefaciens* strain GV3101 pMP90 containing the desired construct was grown overnight in Luria Bertani medium with appropriate antibiotics. A strain carrying a binary vector to express the tomato bushy stunt virus P19 silencing suppressor was cultured in parallel (https://www.plantsci.cam.ac.uk/research/davidbaulcombe/methods/suppressors). Bacterial cells were harvested by centrifugation and resuspended in Infiltration solution (10 mM MgCl2, 10 mM Tris pH 7.5, 100 μM acetosyringone) to A600 of about 1.0. *N*. *benthamiana* leaves (2- to 3-week-old plants, not flowering) were co-infiltrated on the abaxial side with 1 ml of bacterial suspension (1:1 mixture of the strain carrying the desired construct and the strain carrying the P19-expressing plasmid) using a needle-less syringe. After 2–3 days, the infiltrated area was cut out using a cork borer and used for confocal microscopy.

### Bimolecular Fluorescence Complementation (BiFC) assays

Entry clones of full length cDNA sequences without stop codons were used to recombine into the pSPYNE and pSPYCE destination vectors [76] using Gateway LR Clonase II Enzyme mix, to generate BiFC expression constructs. BiFC assays were performed as described previously [20].

### Co-immunoprecipitation

Seedlings of pooled F2 plants from crosses of the parent lines were used to prepare microsomal extracts as described under Western blots. Microsomal pellets were solubilized in PBS + 1.5% Triton X-100 by rotating for 1h at 4°C, and centrifuged for 1h at 13000x g at 4°C to remove the insoluble material. Solubilized microsomes were incubated for 2h at 4°C with α-HA magnetic beads (Pierce Anti-HA magnetic beads, Product #88836 from Thermo Scientific) and then washed with PBS + 1% Triton-X-100 three times. Immunocomplexes were eluted with SDS sample buffer and analyzed by SDS-PAGE. Rat α-HA antibody was used to detect HA-tagged proteins and Mouse α-GFP antibody was used to detect the pulled down PP2CD5:GFP on western blots (S5 Table).

### Western Blots and Far-western blots

Western blot experiments were performed at least twice each. Seedlings grown on MS 1% Sucrose plates in long days for 8 days were used to make protein extracts that were fractionated into Total, Soluble and Microsomal fractions as described in [15]. Equivalent amounts of Total, Soluble and Microsomal protein extracts were subjected to SDS-PAGE. Proteins were transferred to PVDF membranes as recommended by the manufacturers (GE Healthcare). After incubation and washing with the appropriate primary antibody (S5 Table), the membranes were incubated with HRP-linked secondary antibody (S5 Table). After washing off the excess secondary antibody, proteins were revealed with Chemiluminescent detection reagent as recommended by the manufacturers (GE Healthcare) and detected with X-ray film.

14-3-3 overlay far-western blots to detect Thr^947^-phosphorylated H^+^-ATPase used either microsomal extracts as described in [20] or total protein extracts as described in [77] with some modifications. For total protein extract blots, seeds were grown on 0.5X MS 1% Sucrose plates in long days for 5–6 days. Seedlings equivalent to 15 mg in fresh weight were ground with a plastic pestle in 1.5 ml microfuge tubes containing 50 μl of SDS extraction buffer (3% [w/v] SDS, 30 mM Tris-HCl [pH 8.0], 10 mM EDTA, 10 mM NaF, 30% [w/v] Suc, 0.012% [w/v] Coomassie Brilliant Blue, and 15% [v/v] 2-mercaptoethanol). The homogenates were centrifuged at room temperature (10,000x g for 5 min). Aliquots of 10 μl of the supernatant were run on 8% (w/v) SDS-polyacrylamide PAGE gels, and the proteins were transferred to PVDF membranes. The membranes were incubated overnight at 4°C with GST-14-3-3 recombinant fusion protein (purified from an E.coli expression clone, kind gift of T. Kinoshita) at final concentration of 0.1 μM in Blocking buffer (5% dry milk in TBST). Membranes were washed with TBST 3 times, 15 min each, and then incubated with HRP-conjugated anti-GST antibody (Amersham RPN1236) diluted in blocking buffer at 1:15,000, at room temperature for one hour. After washing with TBST, 3 times, 15 min each, SuperSignal West Pico PLUS Chemiluminescent substrate (Thermoscientific) was used for detection. Rabbit α-AHA2-cat antibody (gift of T. Kinoshita) was used at 1:150,000 dilution, and detected with HRP-conjugated Goat anti-rabbit secondary antibody used at 1:30,000.

### Lipid overlay assays

Seedlings were grown in short days for 9 days on MS 1% Sucrose plates, pH 5.7. Total proteins were extracted at 4°C in 500 mM Sucrose; 50 mM Hepes pH 7.4; 5 mM EDTA; 5 mM EGTA; complete protease inhibitor cocktail (Roche). About 40 seedlings were ground with a pestle in 200 μL extraction buffer in a 1.5 ml microfuge tube. Extracts were spun at 13,000x g for 15 min. at 4°C. Protein concentrations of the clear supernatants (Total protein extract) were measured using Protein Assay Dye Reagent Concentrate (BioRad #5000006) according to manufacturer’s instructions. Membrane strips with immobilized purified lipids (PIP strip, P-6001, Echelon Biosciences Inc.) were blocked with TBST (50 mM Tris, 150 mM NaCl, 0.05% Tween-20, pH 7.6) + 3% BSA (Fatty Acid free BSA, Sigma A7030, St. Louis, MO) for 1h at room temperature with shaking; incubated with blocking solution containing about 100 μg of total protein extract for 1h at room temperature with shaking; washed 3 times with TBST; incubated with rabbit anti-HA (Sigma H6908) diluted 1:1000 in Blocking solution; washed 3 times with TBST; incubated 1h with Goat anti-Rabbit IgG-HRP linked (Promega W4011) diluted 1:5000 in Blocking solution; washed 3 times with TBST; and detected by chemiluminescence using ECL as recommended by the manufacturer (GE Healthcare or Thermofisher Scientific).

### Confocal microscopy

To visualize localization of SAUR63:YFP:HA fusion proteins, seedlings were grown in long days (16h day, 8h night) for 5–7 days on vertically-oriented plates with 1X Murashige and Skoog (MS) salts and 0.6% Phyto-agar. Confocal images were taken on a Zeiss LSM 710 DUO confocal microscope with a C-Apochromat 40x W Korr objective. For YFP fluorescence, a 514 nm laser line attenuated to 10% was used for excitation and the capture window was 519–590 nm. To visualize the PM, seedlings were stained with 5 μM FM4-64 (Calbiochem, EMD Millipore, Billerica, MA, USA, Cat #574799, diluted from 10 mM stock into liquid MS medium just before use) at room temperature for 5 minutes in 12 well dishes just before imaging. Fluorescence of FM4-64 was visualized using a 560 nm laser line attenuated to 4.0% for excitation and 601–759 nm for emission. To prevent spectral overlap of FM4-64 and YFP fluorescence, in experiments using FM4-64 staining, YFP was detected using GFP settings of a 488-nm laser line attenuated to 6% for excitation, and 490–535 nm for emission. Non-transgenic wild-type samples were processed in parallel and imaged using the same settings to reveal background fluorescence signals. Root and cotyledon cells were observed in multiple seedlings in at least 2 biological replicate experiments for each genotype. For PAO experiments, a 30 mM stock solution of PAO (Phenyl arsine oxide, Sigma P3075) in DMSO was diluted to 60 μM in liquid MS just before use. Seedlings were treated in 12 well plastic dishes for 30 minutes. PAO experiments were performed between 2 and 9 times for each genotype, and representative images are shown. In image figures, red channel output level representing FM4-64 signal was converted to magenta, and magenta and green channels were adjusted to optimize coincident visibility of both channels.

For 8-Hydroxypyrene-1,3,6-trisulfonic acid (HPTS) staining, seedlings were grown in long days on vertically-oriented 0.5X MS 1% Sucrose plates for 4–5 days. 100 mM HPTS (Sigma-Aldrich H1529) in water was freshly diluted to 1 mM in liquid 0.5X MS just before use. Seedlings were picked off the plate and placed gently in a drop of staining solution on the glass slide. The roots were covered with a coverslip (leaving the cotyledons out) and imaged immediately. Fluorescent signals were collected for the protonated HPTS form (Excitation 405 nm, Emission 500–550 nm), as well as the deprotonated HPTS form (Excitation 458 nm, Emission 500–550 nm) using a water objective (C-Apochromat 40x/1.20 W Korr). Ratiometric images were calculated and analyzed in Fiji (ImageJ) using the macro plug-in described previously [73,78].

### Protein localization in Nicotiana benthamiana or Saccharomyces cerevisiae

Imaging was performed on an inverted Zeiss microscope (AxioObserver Z1, Carl Zeiss Group, http://www.zeiss.com/) equipped with a spinning disk module (CSU-W1-T3, Yokogawa, www.yokogawa.com) and a ProEM+ 1024B camera (Princeton Instrument, http://www.princetoninstruments.com/) using a 63x Plan-Apochromat objective (numerical aperture 1.4, oil immersion). GFP was excited with a 488 nm laser (150mW) and fluorescence emission was filtered by a 525/50 nm single-band bandpass filter (BrightLine, Semrock, http://www.semrock.com/). For the MAP-mCherry:Sac1 experiments in Agrobacterium-infiltrated *N*. *benthamiana* leaves, cells that expressed both i) either *cYFP*:*P4M* PI(4)P-binding control [46,50] or *SAUR63*:*YFP*:*HA*, and ii) active or inactive *MAP-mCherry*:*Sac1* [79]were identified, and scored for putative endomembrane labeling (presence of cytosolic speckles) vs. only PM labeling in confocal image Z-stack projections that spanned the depth of the targeted epidermal cell excluding the upper and lower surfaces (1 μm z-stack intervals, 23–55 images depending on shape and thickness of the cell) [46]. For expression in wild-type or *cho1* mutant yeast, the SAUR63 coding sequence was cloned into the gateway destination vector pAG425GPD-ccdb-eGFP (Addgene #14202) to make a SAUR63:eGFP fusion construct. Protein expression and localization experiments in yeast were done as described in [46]. *cho* mutant yeast cells were generally larger than wild-type cells, and some especially large mutant cells having large intracellular aggregates of SAUR63:GFP fluorescent protein were excluded from the tally. Repeats of these experiments are included in the corresponding figures.

### Statistics

Biological replicate experiments were performed on different dates with freshly germinated seeds. Within each experiment, sample sizes were not computed before doing experiments, but were rather determined by the number of seedlings that could conveniently be grown and measured on petri dishes with two genotypes plated on each (for growth experiments), or by the number of samples that could be processed in the time available (for confocal microscopy experiments). Tukey’s Honestly Significant Difference was calculated from least-squares means. Statistical analyses were performed using JMP 14.0 or 15.0 software (SAS, Cary, NC). Raw data underlying graphs shown in this paper are provided at [80]

## Supporting information

S1 Table Computational analyses of SAUR63 N-terminal domain. (XLSX)

S2 Table NAAIRS mutants of SAUR63 and root tortuosity index. (XLSX)

S3 Table Primers used. (XLSX)

S4 Table Clones for transformation. (XLSX)

S5 Table Antibodies. (XLSX)

S1 Fig Effect of mutating the SAUR63 clade. **A)** Genomic map showing positions of genes and locations of mutations from CRISPR/Cas9 mutagenesis in the *9x-saur* mutant based on the TAIR10 Arabidopsis genome annotation. The first sgRNA directed cuts in both *SAUR61* and *SAUR64* (green arrows), creating a deletion between them (green bar) and leaving behind a hybrid gene with a frameshift at the junction site (symbolized by a green X). The second sgRNA directed cuts in the remaining genes (blue arrows, with lighter blue indicating slight mismatches between the sgRNA and the genome), leading to deletions (blue bars) and/or frameshift mutations (blue X’s). *SAUR* gene names are abbreviated as *S61* etc. *SAUR61*-*SAUR68* are on chromosome 1 and SAUR75 is on chromosome 5. **B,C)** 5-day-old seedlings grown on 1x MS/1% Suc medium in long days. Scale bar, 1 mm. **D)** Hypocotyl lengths of seedlings grown for 4d in short days on 0.5x MS medium. n, 27 (wild type), 22 (*9x-saur*). **E)** Cotyledon area of seedlings grown on vertically oriented plates for 6d on MS/1% Suc medium. n, 16 (wild type), 22 (*9x-saur*). Graphs show means ± s.d. No statistical differences were detected between wild-type and *9x-saur* mutant measurements by t-test. **F)** Sequences of guide RNAs used for CRISPR/Cas9-mediated mutagenesis, wild-type genes, and mutant alleles present in the *9x-saur* mutant. Underlines indicate PAM motif adjacent to guide RNA target site, and any mismatches to the guide RNA sequence. Uppercase bold letters indicate insertion mutations. All alleles create frameshift mutations except for *saur75-1*, which has an in-frame deletion of 13 amino acids in the SAUR domain.
(TIF)

S2 Fig Hypocotyl epidermal cell sizes. **A-F)** Each point represents the length (A, C, E) or width (B, D, F) of a single epidermal cell against distance of that cell from the base of the hypocotyl (x-axis). Seedlings were grown in short days for 2 (A, B), 3 (C, D) or 4 (E, F) days. Cells were measured from confocal z-stack projections using *ML1*:*RFP* PM-localized fluorescence (examples in S3G, S3H and S3I Fig). Data are from 3 cell files per seedling, for two seedlings per time point, except that wild type at 4 days and *ost2-2* at 3 days data are from just one seedling each. In a replicate experiment with 2-day-old seedlings only, *P*_*35S*_:*SAUR63*:*YFP*:*HA* hypocotyls also had a greater proportion of longer cells than did wild-type hypocotyls.
(TIF)

S3 Fig Seedling phenotypes of plants expressing SAUR63:X fusion proteins. **A,B)** Hypocotyl length (A) and hypocotyl tortuosity index [B, 1 –(distance between ends)/(contour length)]) of seedlings of indicated genotypes grown for 3d in darkness on plates with 0.5x MS medium. **C,D)** Root length (C) and root tortuosity index (D) of seedlings of indicated genotypes grown for 4d in long days on plates with 1x MS 1% Sucrose medium. **E,F)** Root length (E) and root tortuosity index (F) of seedlings of indicated genotypes grown for 4d in short days on plates with 0.5x MS medium. Graphs show means ± s.d. Letters in graphs indicate statistical classes based on Tukey’s Honestly Significant Difference test. n from left to right: Panels A,B: 51, 24, 26, 28, 23, 25, 25; Panels C,D: 34, 17, 23, 14, 19, 15, 16; Panels E,F: 43, 21, 18, 17, 16, 15, 17. The same genotypes were measured in panels A-F, with genotype designations shown only in panels E and F. **G,H,I)** Hypocotyl epidermal cells of seedlings of indicated genotypes grown in short days for 2 days, visualized with the *ML1*:*RFP* shoot epidermis plasma membrane marker. Shown are z-stack confocal image projections of the near side of the hypocotyl. Scale bar, 0.1 mm. S2 Fig shows measurements of cell sizes in this experiment.
(TIF)

S4 Fig Seedling phenotypes of plants expressing SAUR63:YFP:HA variants. **A)** Cotyledon area of seedlings of indicated genotypes grown on vertically oriented plates for 7d in long days in the absence (open bars) or presence (filled bars) of 1% sucrose. **B)** Root length of seedlings of indicated genotypes grown on vertically oriented plates for 4d in long days in the absence (open bars) or presence (filled bars) of 1% sucrose. **C,F)** Hypocotyl lengths (E) and hypocotyl tortuosity index [F, 1 –(distance between ends)/(contour length)] of seedlings grown on vertically oriented plates for 3d in darkness without sucrose. **D)** Cotyledon areas of *P*_*35S*_:*SAUR63* and *P*_*35S*_:*CBL1*^*1-12*^:*SAUR63*^*26-142*^ seedlings grown for 6d on vertically oriented MS 1% Suc plates. **E)** Hypocotyl lengths of *P*_*35S*_:*SAUR63* and *P*_*35S*_:*CBL1*^*1-12*^:*SAUR63*^*26-142*^ seedlings grown for 4d on vertically oriented 0.5x MS plates. Panels D and E show data for the three homozygous single-locus *P*_*35S*_:*SAUR63* lines that differed most from wild type among seven lines analyzed. Graphs show means ± s.d. Letters in graphs indicate statistical classes based on Tukey’s Honestly Significant Difference test. n, from left to right: panel A: 26, 20, 25, 15, 22, 23, 24, 20, 22, 23, 19, 19, 21, 19, 21, 20; panel B: 19, 16, 18, 14, 20, 20, 21, 16, 21, 17, 19, 16, 18, 15, 16, 17; panel D: 77, 18, 17, 17, 19, 20; panel E: 119, 25, 24, 27, 21, 24; panels C and F: 35, 36, 45, 43, 47, 38, 41, 47.
(TIF)

S5 Fig Epistasis among SAUR63 and PP2C.D5 lines, and phenotypes related to PM H^+^-ATPase activity. **A)** Appearance of genotypes used for crosses presented in A and in Fig 2C and 2D, grown for 6d in long days in the presence of sucrose. Scale bar, 3 mm. **B)** Hypocotyl lengths of seedlings of indicated genotypes grown for 4d in short days in the absence of sucrose. Plants measured were F1 progeny of crosses of transgenic lines with each other or with wild-type Columbia, and were hemizygous for the indicated transgene(s). Fig 2C shows a subset of this data. A replicate experiment gave similar results. **C)** Appearance of *P*_*EST*_:*SAUR63*:*CerFP*:*HA* and wild-type seedlings grown with estradiol and in the absence or presence of 15 mM LiCl. Seedlings were grown for 3d under control conditions, and then transferred to plates with estradiol and with or without 15 mM LiCl, and grown for an additional 3d before imaging. Dots mark positions of root tips at the time of transfer to estradiol plates. Scale bar, 5 mm. **D)** Root growth of indicated genotypes in the absence (open bars) or presence (closed bars) of 15 mM LiCl. Seedlings were grown without LiCl for 5d, transferred to plates containing 0 or 15 mM LiCl, and root growth over the next three days was measured. **E)** HPTS fluorescence ratios around root cells of indicated genotypes. Data are pooled from measurements taken on three different days, each normalized to the average of wild-type values on those days. Graphs show means ± s.d. Letters in graphs indicate statistical classes based on Tukey’s Honestly Significant Difference test. n, from left to right: panel B: 28, 24, 24, 30, 26, 27, 26, 25, 26, 25, 24, 20, 24, 26, 25; panel D: 21, 27, 16, 23, 19, 25, 20, 30, 18, 28, 21, 30, 23, 31, 18, 29; panel E: 26, 13, 14, 16.
(TIF)

S6 Fig Localization of SAUR63:YFP:HA in root meristem epidermal cells compared to control WAVE lines. **A-D)** *P*_*35S*_:*SAUR63*:*YFP*:*HA*. **E-H)** *P*_*35S*_:*SAUR63*^*26-142*^:*YFP*:*HA*. **I-L)** *P*_*UBQ10*_:*WAVE 138Y* expressing a PM-localized YFP fusion protein. **M-P)** *P*_*UBQ10*_:*WAVE 1Y* expressing a cytoplasmically-localized YFP fusion protein. **Q-T)** *P*_*UBQ10*_:*WAVE 9Y* expressing a YFP fusion protein localized to the tonoplast. Shown are fluorescence confocal microscopy images of YFP (green, A,E,I,M,Q), FM4-64 membrane staining (magenta, B,F,J,N,R), and both channels together (C,G,K,O,S) with vertical yellow lines indicating locations of quantitation of fluorescence intensity signals, scaled to the maximum signal along the line (D,H,L,P,T). Image color channel brightnesses were adjusted for visibility. Scale bar, 20 μm.
(TIF)

S7 Fig Visualization of SAUR63:YFP:HA fusion protein variants in cotyledons. **A-M)** Confocal images showing YFP fluorescence of transgenic lines expressing the indicated fusion proteins behind the *P*_*35S*_ promoter. Scale bar, 20 μm.
(TIF)

S8 Fig Localization of SAUR63:YFP:HA variants in *Nicotiana benthamiana* leaf cells. **A-L)** Confocal images showing YFP fluorescence of indicated SAUR63:YFP:HA variants expressed in transiently transformed *N*. *benthamiana* leaves. Scale bar, 20 μm.
(TIF)

S9 Fig Effects of N-terminal alterations on protein levels and fractionation of SAUR63:YFP:HA fusion proteins. **A-E)** Protein levels in multiple *P*_*35S*_:*SAUR63*:*YFP*:*HA*, *P*_*35S*_:*SAUR63*^*26-142*^:*YFP*:*HA*, and *P*_*35S*_:*CBL1*^*1-12*^:*SAUR63*^*26-142*^:*YFP*:*HA* pooled T2 seedlings from different T1 transformants. The blot in panel E shows protein extracts from selected genotypes in panels A-D for side-by-side comparison in the same experiment. **F,G)** SAUR63:YFP:HA, SAUR63^26-142^:YFP:HA, SAUR63^1-25^:YFP:HA, CBL1^1-12^:SAUR63^26-142^:YFP:HA, and SAUR63^m2^:YFP:HA fusion proteins, detected by western blots in total (T), soluble (S) and microsomal (M) protein fractions. Lower panels show controls for loading or fractionation. α-HA detects SAUR63 fusion protein; α-AHA2 detects a membrane protein; α-UGPase and α-APX detect soluble proteins. Arrows indicate position of full-sized SAUR63:YFP:HA fusion proteins. FL, Full-length SAUR63:YFP:HA fusion protein. wt, wild-type Columbia lacking any transgene. In genotype designations, S63 is short for SAUR63. Letters and numbers after genotype designations indicate independent transgenic lines. **H)** Amount of SAUR63:YFP:HA and SAUR63^26-142^:YFP:HA proteins in whole seedling extracts at indicated times after start of cycloheximide treatment to block new protein synthesis. Fusion proteins were detected by western blots using anti-HA antibody. The Rubisco large subunit band from Ponceau S staining of the same gels is shown as a loading control. A repeat of this experiment gave a very similar result. In the lower gel, the larger band is the presumed intact SAUR63^26-142^:YFP:HA protein, and the lower band is a presumed smaller breakdown product. In genotype labels, S63 denotes SAUR63, letters and numbers after genotype names indicate distinct transgenic lines, FL denotes a strong full-length *P*_*35S*_:*SAUR63*:*YFP*:*HA* line used as a common standard line in most experiments, and wt indicates wild-type Columbia lacking any transgene.
(TIF)

S10 Fig SAUR63 fusion protein lipid binding. **A)** Western blot showing presence of fusion proteins in extracts used in lipid blot experiments in Fig 5A. Arrows indicate locations of full-length SAUR63:YFP:HA and truncated SAUR63^26-142^:YFP:HA fusion proteins (upper arrow) and SAUR63^1-25^:YFP:HA N-terminal domain fusion protein (lower arrow). **B)** Longer exposures of two lipid blots from Fig 5A. **C)** Mock experiment in which extracts were incubated for 70 minutes in protein extraction buffer at the indicated temperatures, and then run on a gel for western blots. For both SAUR63:YFP:HA and SAUR63^26-142^:YFP:HA, similar amounts of protein are present after incubation at -20 C or after incubation at 22 C, as during the lipid blot binding experiment.
(TIF)

S11 Fig *P*_*EST*_:*SAUR63*^*NAAIRS*^:*CerFP*:*HA* lines. **A)** *P*_*EST*_:*SAUR63*^*NAAIRS*^:*CerFP*:*HA* lines grown on plates with estradiol (all genotypes) or without estradiol (wild-type Columbia and *P*_*EST*_:*SAUR63*:*CerFP*:*HA* only). S2 Table indicates amino acids changed in each mutant and root tortuosity index measurement data from this and one replicate experiment. Scale bar, 1 cm. **B)** Western blots of total protein in estradiol-induced *P*_*EST*_:*SAUR63*^*NAAIRS*^:*CerFP*:*HA* lines using α-HA antibody.
(TIF)

S12 Fig Effects of mutations on SAUR63:YFP:HA localization in root meristem cells. **A-D)** *P*_*35S*_:*SAUR63*:*YFP*:*HA*. **E-H)** *P*_*35S*_:*SAUR63*^*26-142*^:*YFP*:*HA*. **I-L)** *P*_*35S*_:*SAUR63*^*m9*^:*YFP*:*HA*. **M-P)** *P*_*35S*_:*SAUR63*^*m13*^:*YFP*:*HA*. **Q-T)** *P*_*35S*_:*SAUR63*^*m15*^:*YFP*:*HA*. Shown are fluorescence confocal microscopy images of YFP (green, A,E,I,M,Q), FM4-64 membrane staining (magenta, B,F,J,N,R), and both channels together (C,G,K,O,S) with vertical yellow lines indicating locations of quantitation of fluorescence intensity signals, scaled to the maximum signal along the line (D,H,L,P,T). Image color channel brightnesses were adjusted for visibility. Panels A-D are the same as in Fig 8. Scale bar, 20 μm.
(TIF)
