# Supplementary figures and images for: SAUR63 stimulates cell growth at the plasma membrane
Source: PLoS Genet. 2022 Sep 19;18(9):e1010375. doi: 10.1371/journal.pgen.1010375 (PMC9522268; doi:10.1371/journal.pgen.1010375)

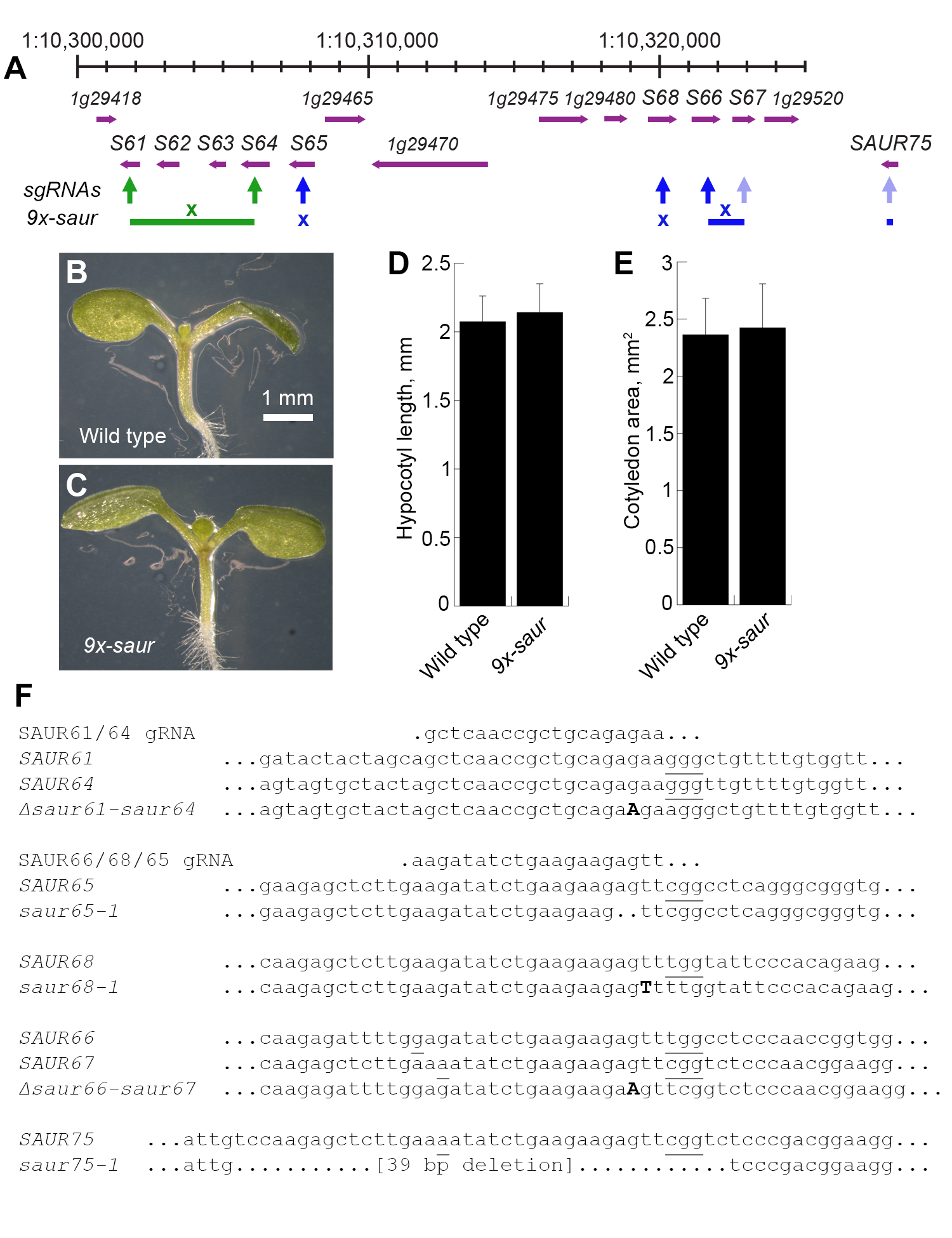

Supplement: S1 Fig — A) Genomic map showing positions of genes and locations of mutations from CRISPR/Cas9 mutagenesis in the 9x-saur mutant based on the TAIR10 Arabidopsis genome annotation. The first sgRNA directed cuts in both SAUR61 and SAUR64 (green arrows), creating a deletion between them (green bar) and leaving behind a hybrid gene with a frameshift at the junction site (symbolized by a green X). The second sgRNA directed cuts in the remaining genes (blue arrows, with lighter blue indicating slight mismatches between the sgRNA and the genome), leading to deletions (blue bars) and/or frameshift mutations (blue X’s). SAUR gene names are abbreviated as S61 etc. SAUR61-SAUR68 are on chromosome 1 and SAUR75 is on chromosome 5. B,C) 5-day-old seedlings grown on 1x MS/1% Suc medium in long days. Scale bar, 1 mm. D) Hypocotyl lengths of seedlings grown for 4d in short days on 0.5x MS medium. n, 27 (wild type), 22 (9x-saur). E) Cotyledon area of seedlings grown on vertically oriented plates for 6d on MS/1% Suc medium. n, 16 (wild type), 22 (9x-saur). Graphs show means ± s.d. No statistical differences were detected between wild-type and 9x-saur mutant measurements by t-test. F) Sequences of guide RNAs used for CRISPR/Cas9-mediated mutagenesis, wild-type genes, and mutant alleles present in the 9x-saur mutant. Underlines indicate PAM motif adjacent to guide RNA target site, and any mismatches to the guide RNA sequence. Uppercase bold letters indicate insertion mutations. All alleles create frameshift mutations except for saur75-1, which has an in-frame deletion of 13 amino acids in the SAUR domain. (TIF) [file pgen.1010375.s006.tif]

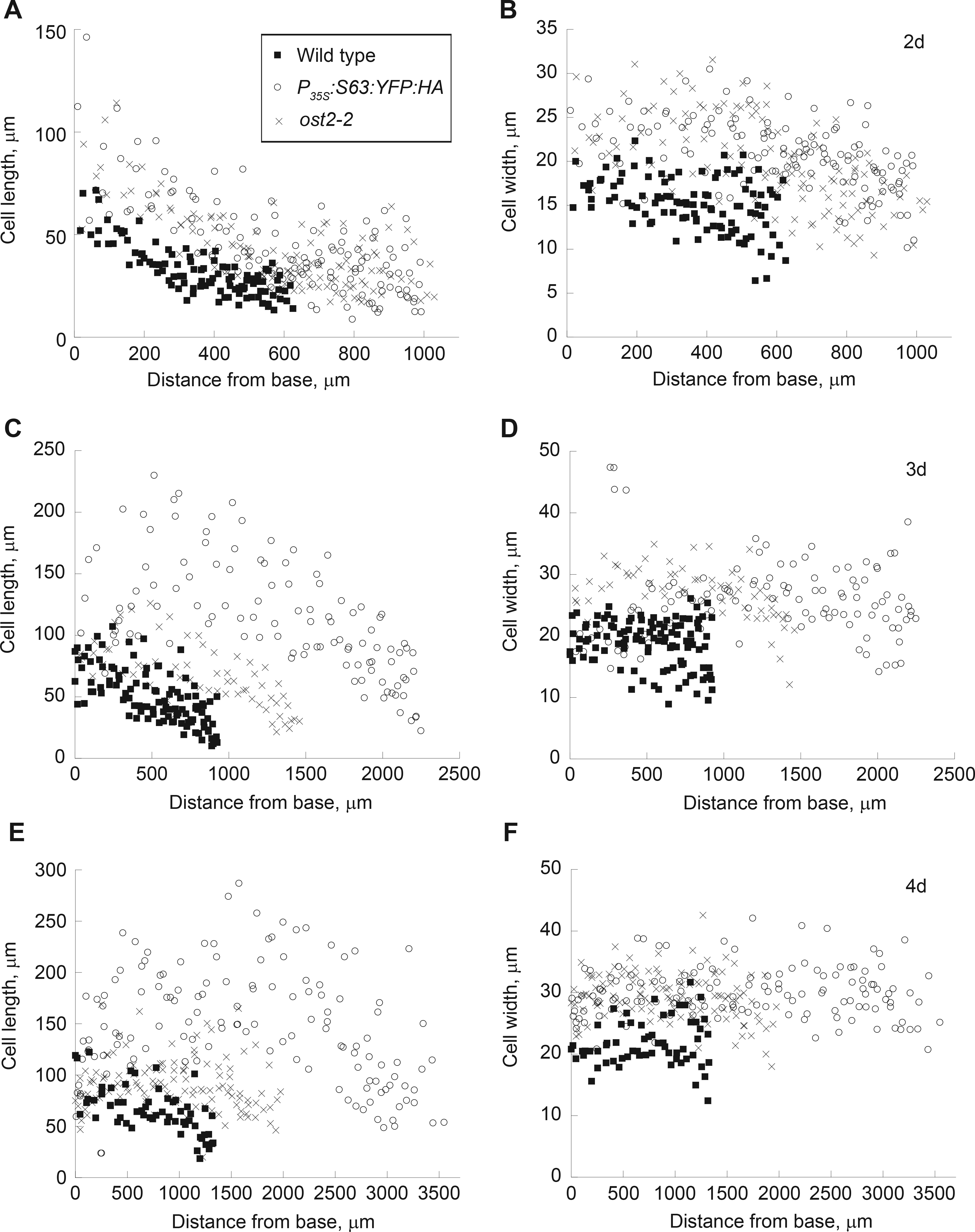

Supplement: S2 Fig — A-F) Each point represents the length (A, C, E) or width (B, D, F) of a single epidermal cell against distance of that cell from the base of the hypocotyl (x-axis). Seedlings were grown in short days for 2 (A, B), 3 (C, D) or 4 (E, F) days. Cells were measured from confocal z-stack projections using ML1:RFP PM-localized fluorescence (examples in S3G, S3H and S3I Fig). Data are from 3 cell files per seedling, for two seedlings per time point, except that wild type at 4 days and ost2-2 at 3 days data are from just one seedling each. In a replicate experiment with 2-day-old seedlings only, P35S:SAUR63:YFP:HA hypocotyls also had a greater proportion of longer cells than did wild-type hypocotyls. (TIF) [file pgen.1010375.s007.tif]

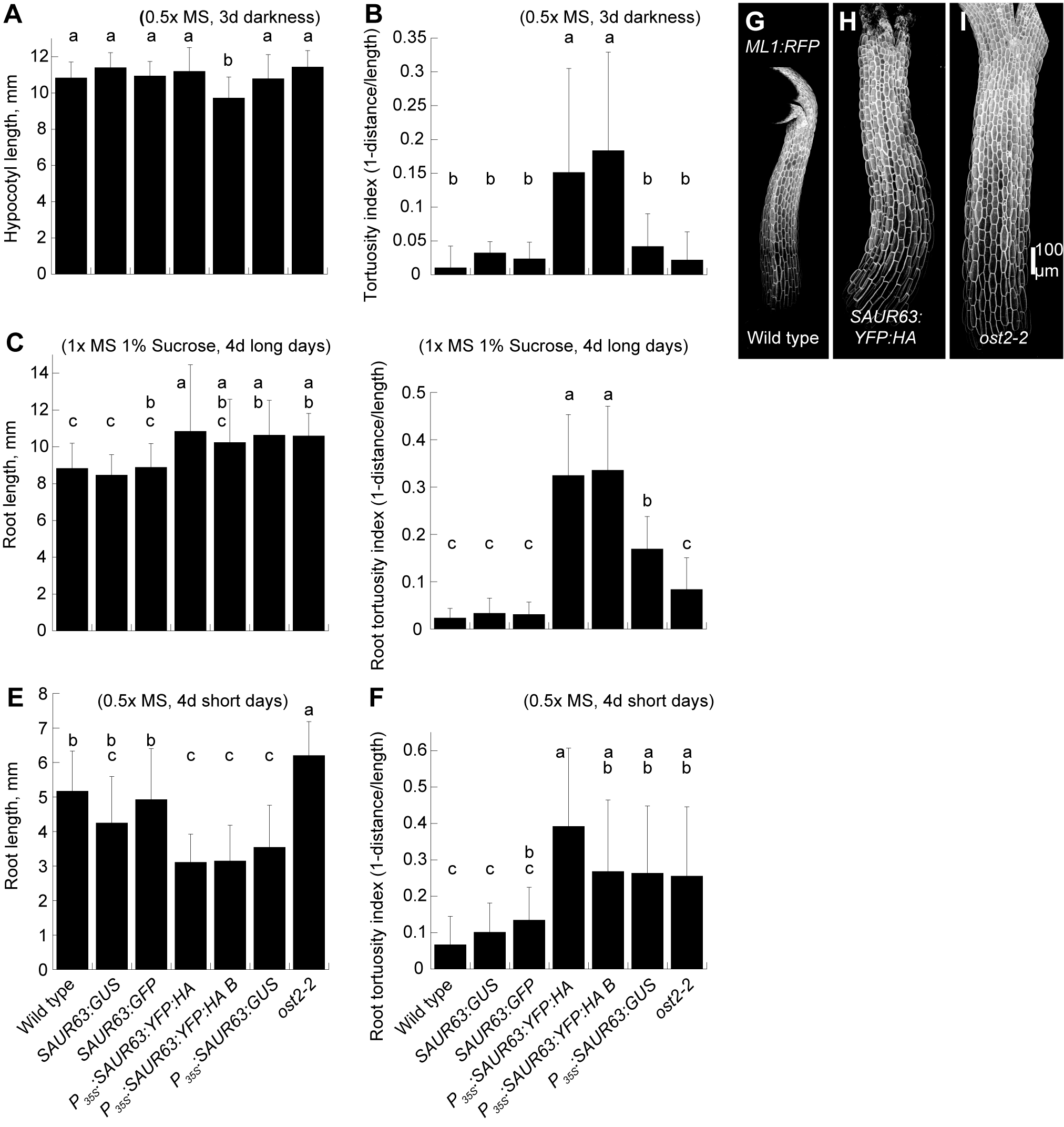

Supplement: S3 Fig — A,B) Hypocotyl length (A) and hypocotyl tortuosity index [B, 1 –(distance between ends)/(contour length)]) of seedlings of indicated genotypes grown for 3d in darkness on plates with 0.5x MS medium. C,D) Root length (C) and root tortuosity index (D) of seedlings of indicated genotypes grown for 4d in long days on plates with 1x MS 1% Sucrose medium. E,F) Root length (E) and root tortuosity index (F) of seedlings of indicated genotypes grown for 4d in short days on plates with 0.5x MS medium. Graphs show means ± s.d. Letters in graphs indicate statistical classes based on Tukey’s Honestly Significant Difference test. n from left to right: Panels A,B: 51, 24, 26, 28, 23, 25, 25; Panels C,D: 34, 17, 23, 14, 19, 15, 16; Panels E,F: 43, 21, 18, 17, 16, 15, 17. The same genotypes were measured in panels A-F, with genotype designations shown only in panels E and F. G,H,I) Hypocotyl epidermal cells of seedlings of indicated genotypes grown in short days for 2 days, visualized with the ML1:RFP shoot epidermis plasma membrane marker. Shown are z-stack confocal image projections of the near side of the hypocotyl. Scale bar, 0.1 mm. S2 Fig shows measurements of cell sizes in this experiment. (TIF) [file pgen.1010375.s008.tif]

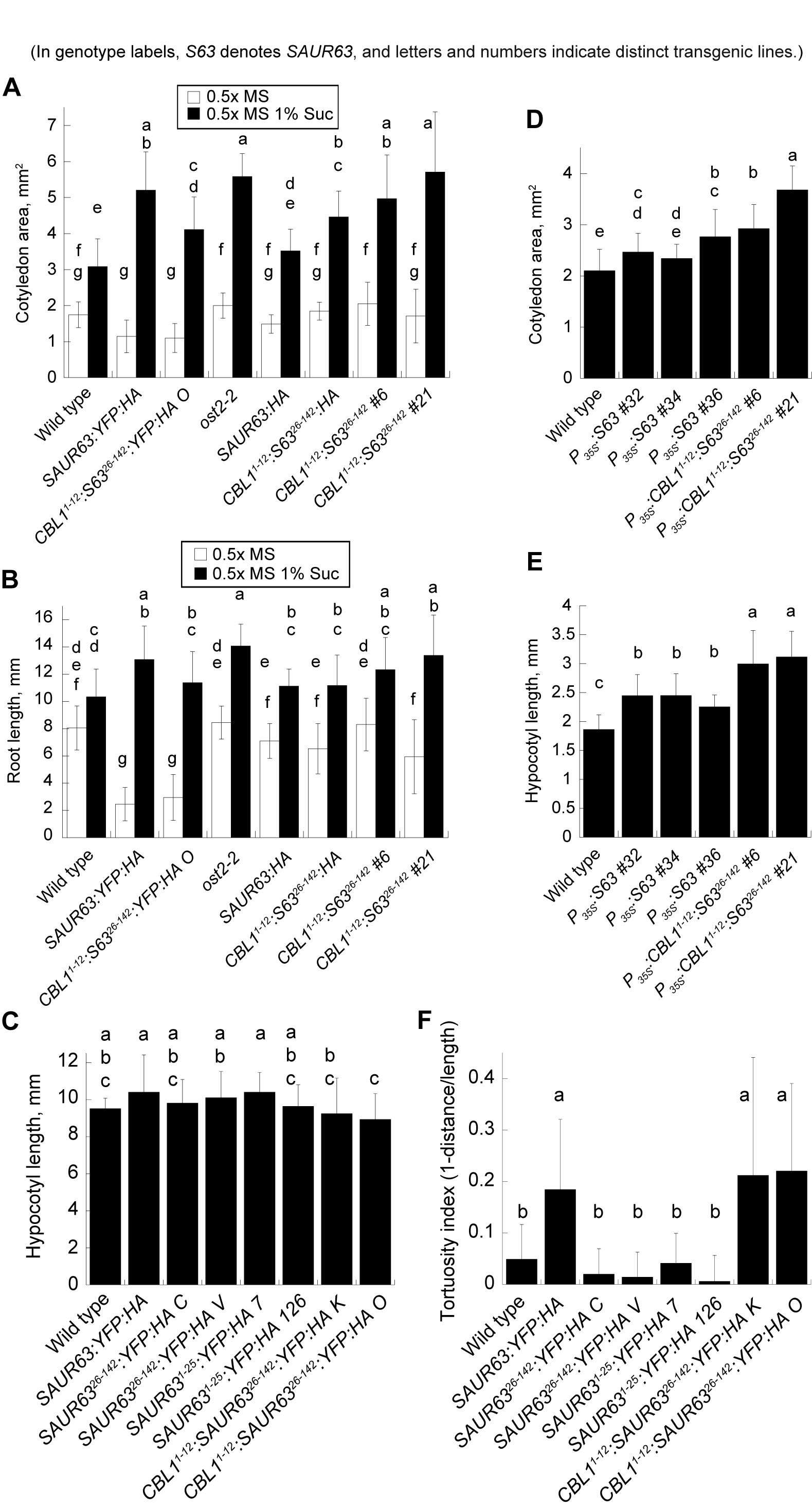

Supplement: S4 Fig — A) Cotyledon area of seedlings of indicated genotypes grown on vertically oriented plates for 7d in long days in the absence (open bars) or presence (filled bars) of 1% sucrose. B) Root length of seedlings of indicated genotypes grown on vertically oriented plates for 4d in long days in the absence (open bars) or presence (filled bars) of 1% sucrose. C,F) Hypocotyl lengths (E) and hypocotyl tortuosity index [F, 1 –(distance between ends)/(contour length)] of seedlings grown on vertically oriented plates for 3d in darkness without sucrose. D) Cotyledon areas of P35S:SAUR63 and P35S:CBL11-12:SAUR6326-142 seedlings grown for 6d on vertically oriented MS 1% Suc plates. E) Hypocotyl lengths of P35S:SAUR63 and P35S:CBL11-12:SAUR6326-142 seedlings grown for 4d on vertically oriented 0.5x MS plates. Panels D and E show data for the three homozygous single-locus P35S:SAUR63 lines that differed most from wild type among seven lines analyzed. Graphs show means ± s.d. Letters in graphs indicate statistical classes based on Tukey’s Honestly Significant Difference test. n, from left to right: panel A: 26, 20, 25, 15, 22, 23, 24, 20, 22, 23, 19, 19, 21, 19, 21, 20; panel B: 19, 16, 18, 14, 20, 20, 21, 16, 21, 17, 19, 16, 18, 15, 16, 17; panel D: 77, 18, 17, 17, 19, 20; panel E: 119, 25, 24, 27, 21, 24; panels C and F: 35, 36, 45, 43, 47, 38, 41, 47. (TIF) [file pgen.1010375.s009.tif]

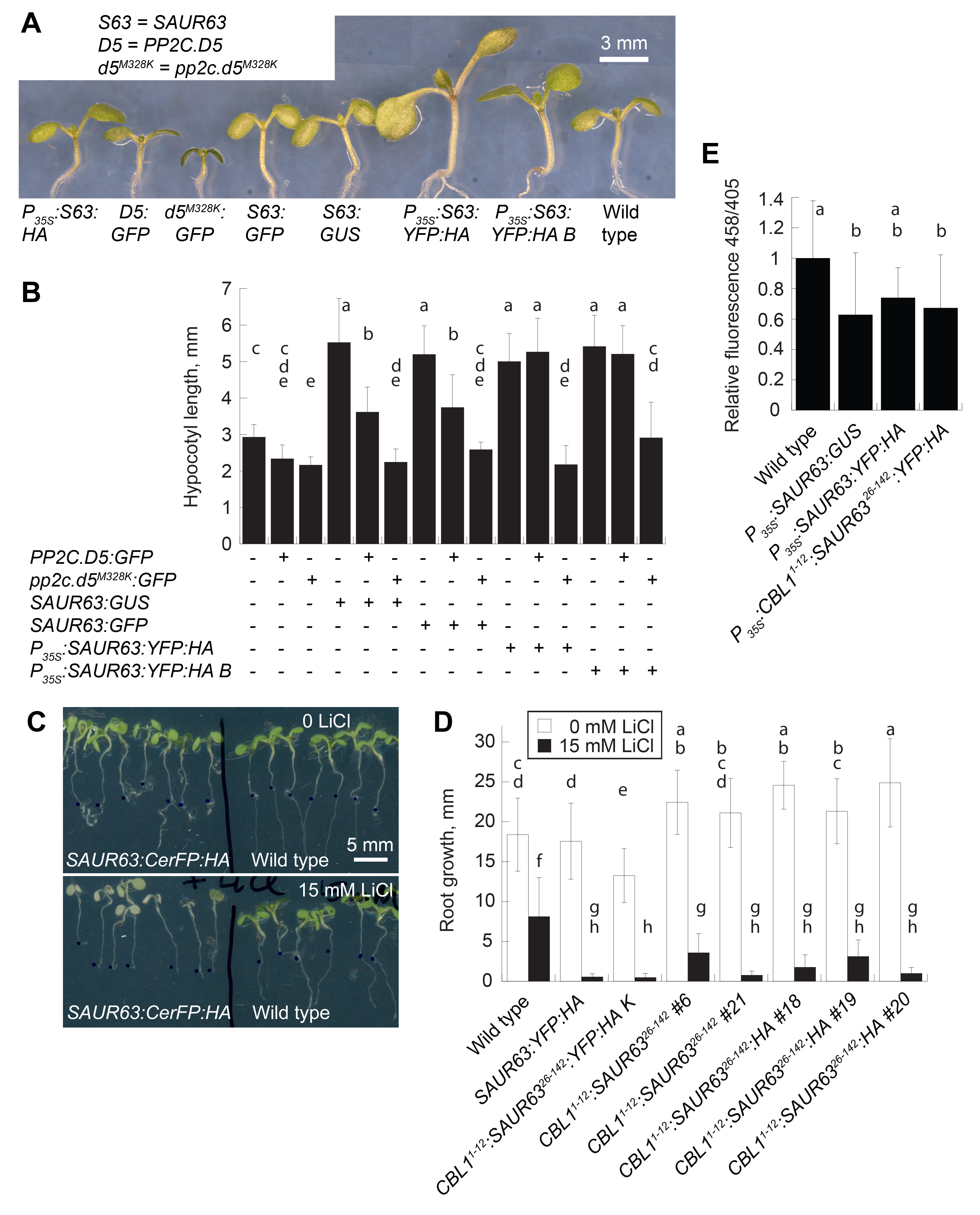

Supplement: S5 Fig — A) Appearance of genotypes used for crosses presented in A and in Fig 2C and 2D, grown for 6d in long days in the presence of sucrose. Scale bar, 3 mm. B) Hypocotyl lengths of seedlings of indicated genotypes grown for 4d in short days in the absence of sucrose. Plants measured were F1 progeny of crosses of transgenic lines with each other or with wild-type Columbia, and were hemizygous for the indicated transgene(s). Fig 2C shows a subset of this data. A replicate experiment gave similar results. C) Appearance of PEST:SAUR63:CerFP:HA and wild-type seedlings grown with estradiol and in the absence or presence of 15 mM LiCl. Seedlings were grown for 3d under control conditions, and then transferred to plates with estradiol and with or without 15 mM LiCl, and grown for an additional 3d before imaging. Dots mark positions of root tips at the time of transfer to estradiol plates. Scale bar, 5 mm. D) Root growth of indicated genotypes in the absence (open bars) or presence (closed bars) of 15 mM LiCl. Seedlings were grown without LiCl for 5d, transferred to plates containing 0 or 15 mM LiCl, and root growth over the next three days was measured. E) HPTS fluorescence ratios around root cells of indicated genotypes. Data are pooled from measurements taken on three different days, each normalized to the average of wild-type values on those days. Graphs show means ± s.d. Letters in graphs indicate statistical classes based on Tukey’s Honestly Significant Difference test. n, from left to right: panel B: 28, 24, 24, 30, 26, 27, 26, 25, 26, 25, 24, 20, 24, 26, 25; panel D: 21, 27, 16, 23, 19, 25, 20, 30, 18, 28, 21, 30, 23, 31, 18, 29; panel E: 26, 13, 14, 16. (TIF) [file pgen.1010375.s010.tif]

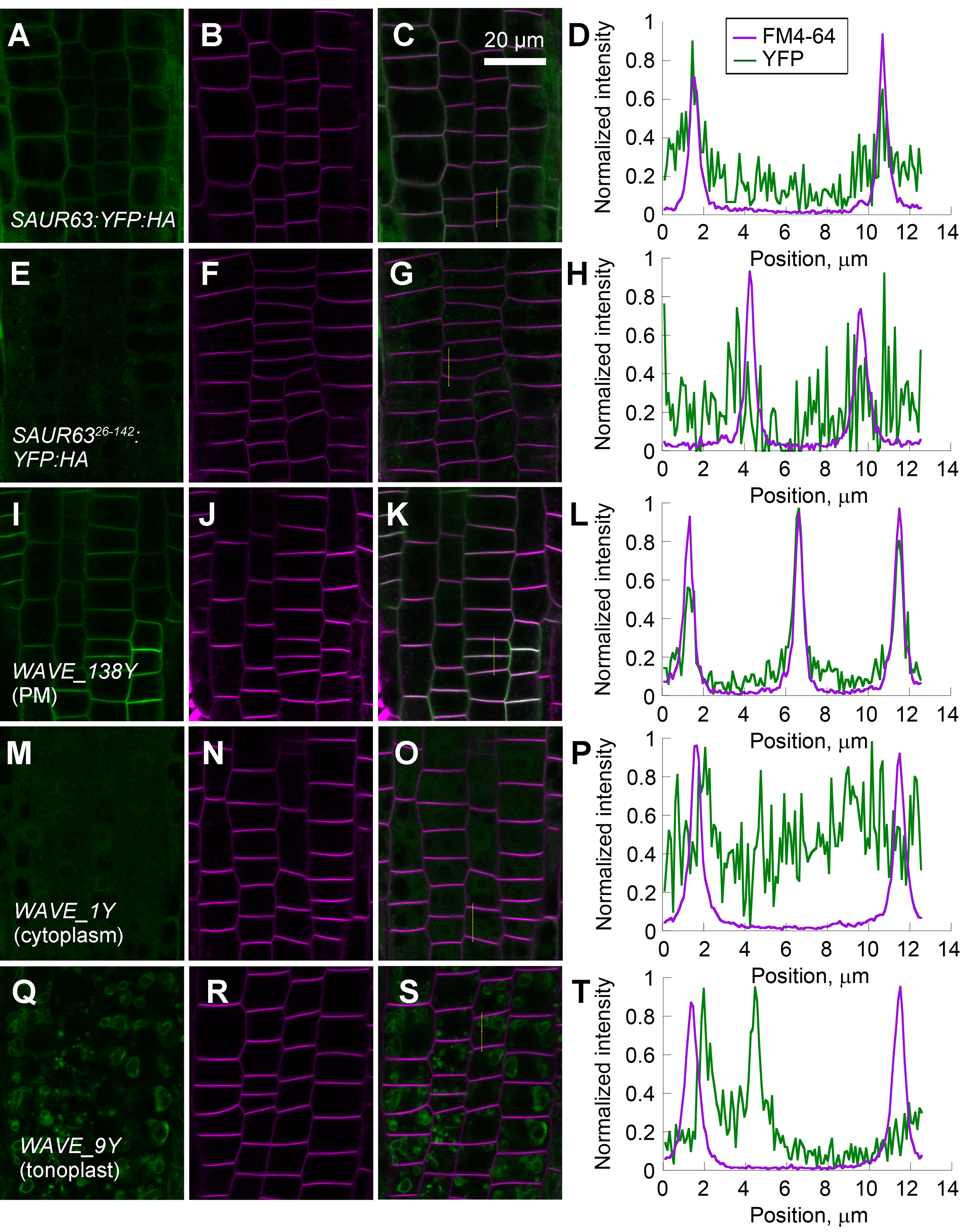

Supplement: S6 Fig — A-D) P35S:SAUR63:YFP:HA. E-H) P35S:SAUR6326-142:YFP:HA. I-L) PUBQ10:WAVE 138Y expressing a PM-localized YFP fusion protein. M-P) PUBQ10:WAVE 1Y expressing a cytoplasmically-localized YFP fusion protein. Q-T) PUBQ10:WAVE 9Y expressing a YFP fusion protein localized to the tonoplast. Shown are fluorescence confocal microscopy images of YFP (green, A,E,I,M,Q), FM4-64 membrane staining (magenta, B,F,J,N,R), and both channels together (C,G,K,O,S) with vertical yellow lines indicating locations of quantitation of fluorescence intensity signals, scaled to the maximum signal along the line (D,H,L,P,T). Image color channel brightnesses were adjusted for visibility. Scale bar, 20 μm. (TIF) [file pgen.1010375.s011.tif]

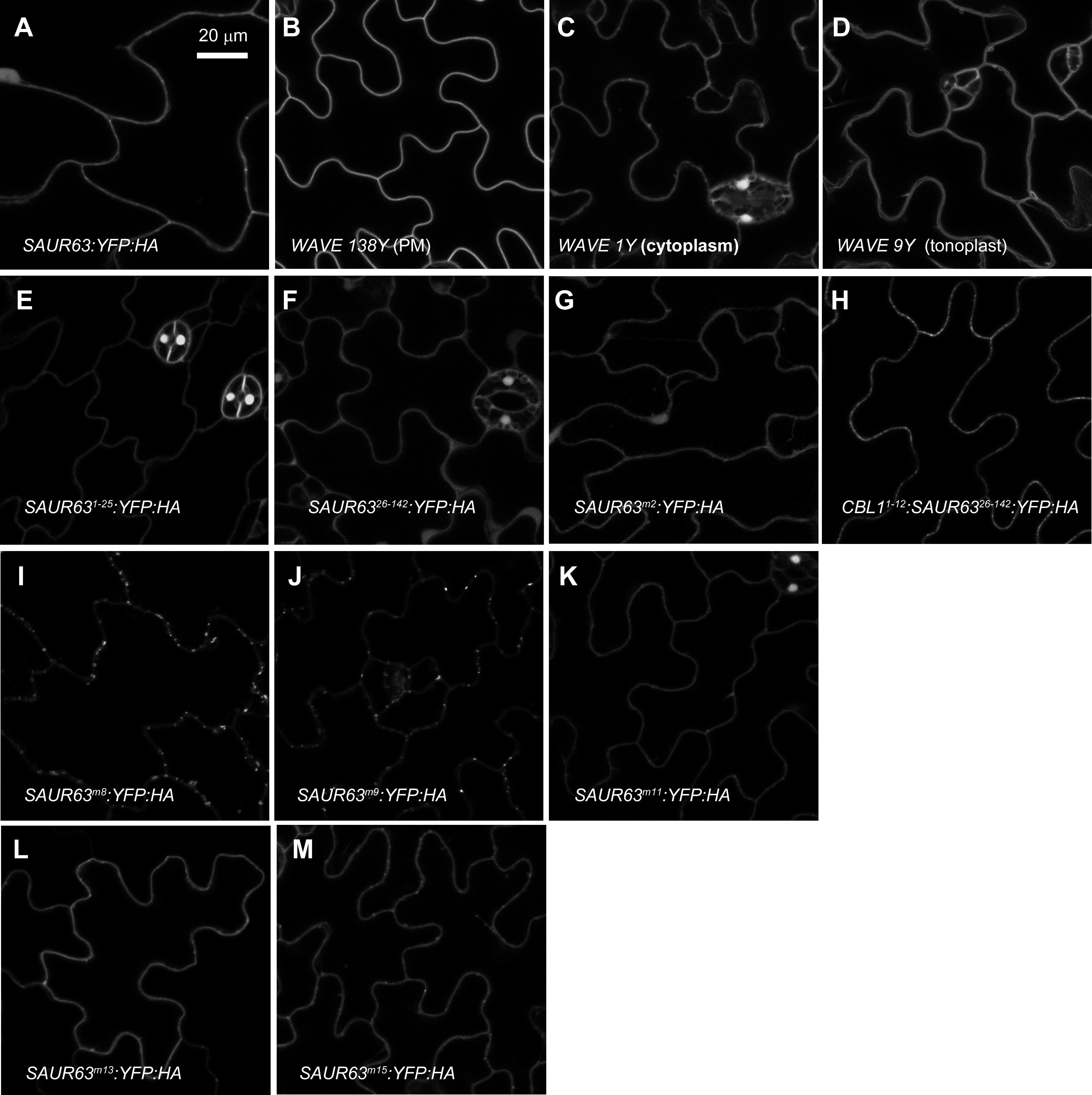

Supplement: S7 Fig — A-M) Confocal images showing YFP fluorescence of transgenic lines expressing the indicated fusion proteins behind the P35S promoter. Scale bar, 20 μm. (TIF) [file pgen.1010375.s012.tif]

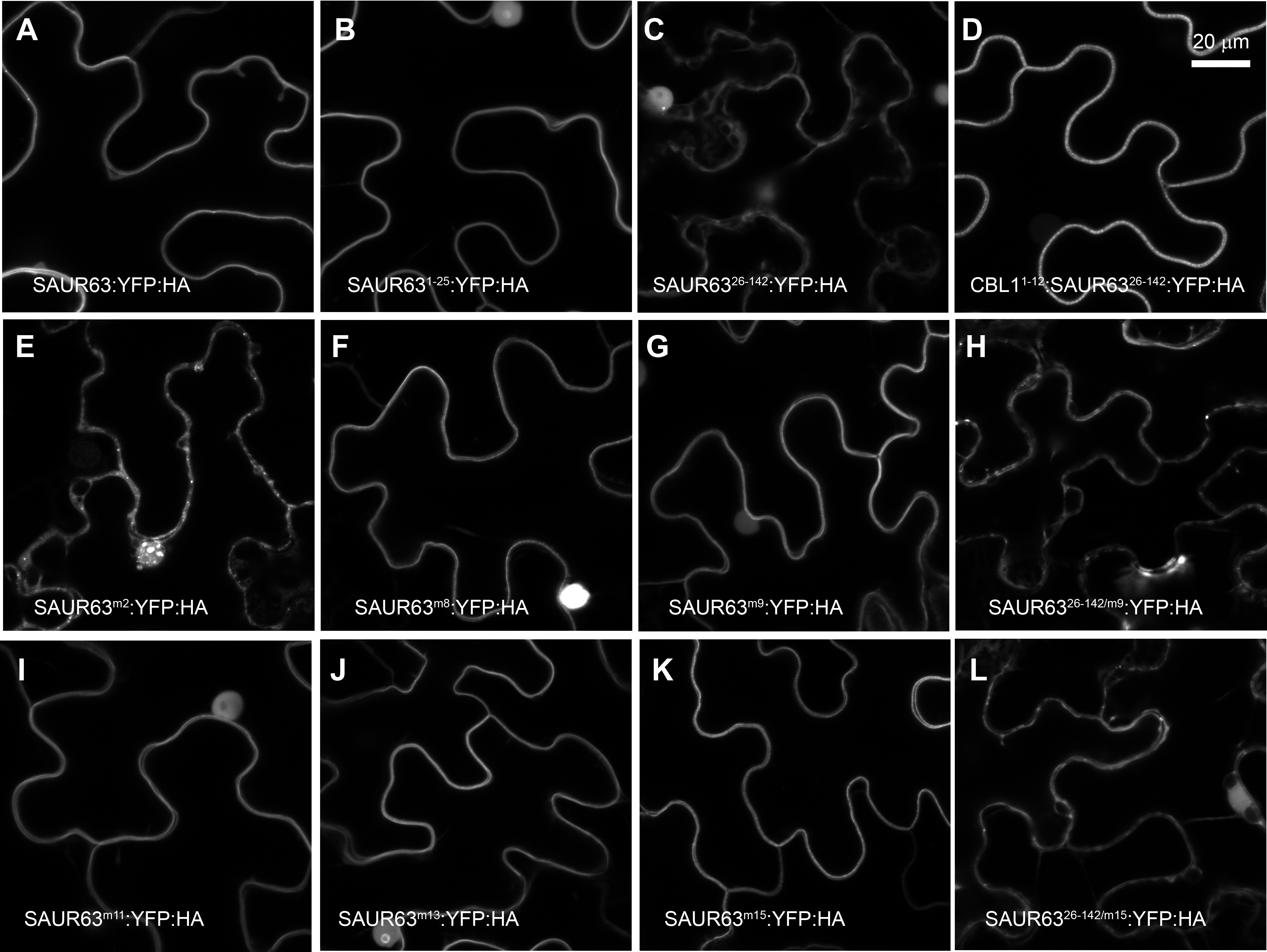

Supplement: S8 Fig — A-L) Confocal images showing YFP fluorescence of indicated SAUR63:YFP:HA variants expressed in transiently transformed N. benthamiana leaves. Scale bar, 20 μm. (TIF) [file pgen.1010375.s013.tif]

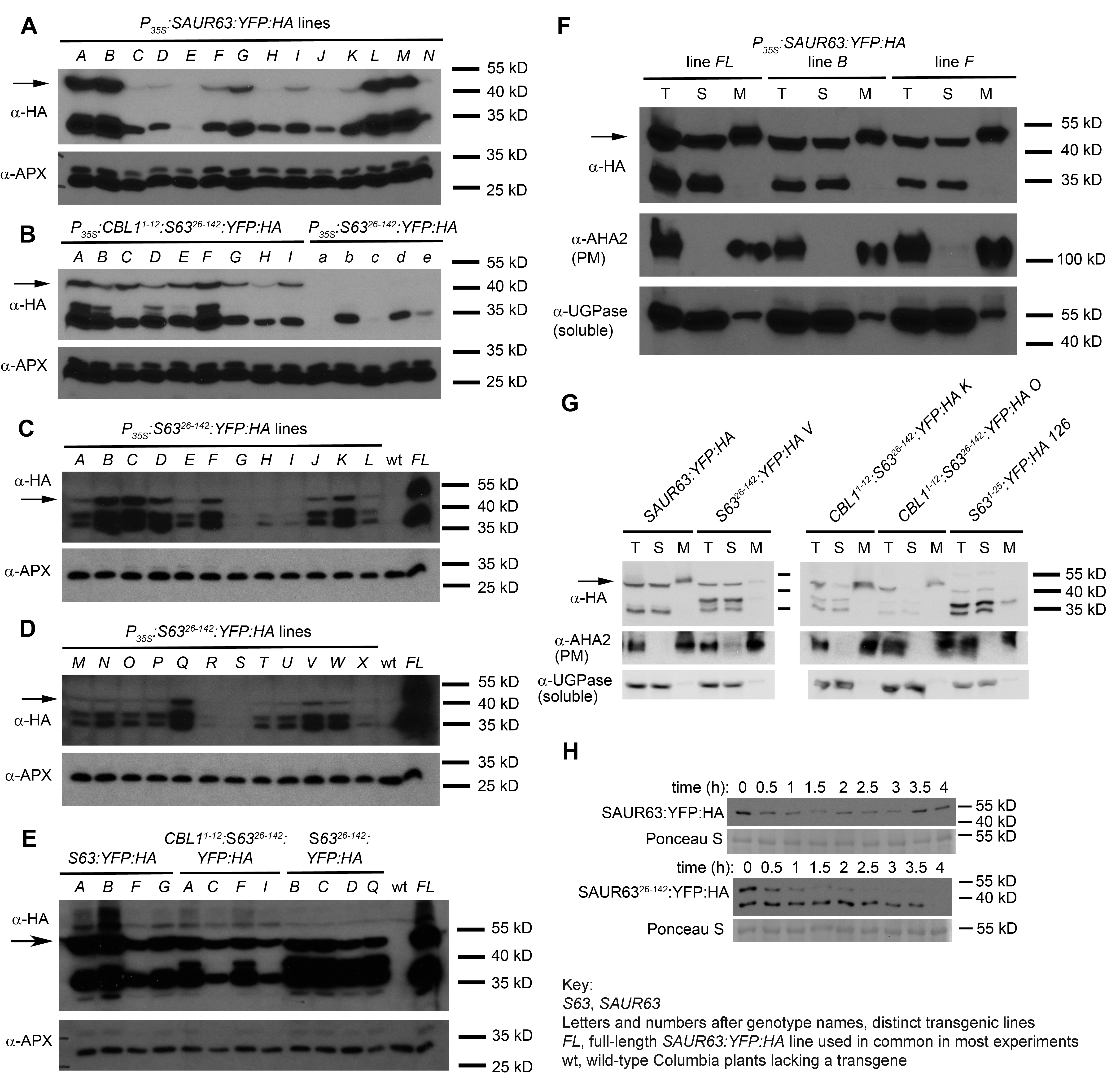

Supplement: S9 Fig — A-E) Protein levels in multiple P35S:SAUR63:YFP:HA, P35S:SAUR6326-142:YFP:HA, and P35S:CBL11-12:SAUR6326-142:YFP:HA pooled T2 seedlings from different T1 transformants. The blot in panel E shows protein extracts from selected genotypes in panels A-D for side-by-side comparison in the same experiment. F,G) SAUR63:YFP:HA, SAUR6326-142:YFP:HA, SAUR631-25:YFP:HA, CBL11-12:SAUR6326-142:YFP:HA, and SAUR63m2:YFP:HA fusion proteins, detected by western blots in total (T), soluble (S) and microsomal (M) protein fractions. Lower panels show controls for loading or fractionation. α-HA detects SAUR63 fusion protein; α-AHA2 detects a membrane protein; α-UGPase and α-APX detect soluble proteins. Arrows indicate position of full-sized SAUR63:YFP:HA fusion proteins. FL, Full-length SAUR63:YFP:HA fusion protein. wt, wild-type Columbia lacking any transgene. In genotype designations, S63 is short for SAUR63. Letters and numbers after genotype designations indicate independent transgenic lines. H) Amount of SAUR63:YFP:HA and SAUR6326-142:YFP:HA proteins in whole seedling extracts at indicated times after start of cycloheximide treatment to block new protein synthesis. Fusion proteins were detected by western blots using anti-HA antibody. The Rubisco large subunit band from Ponceau S staining of the same gels is shown as a loading control. A repeat of this experiment gave a very similar result. In the lower gel, the larger band is the presumed intact SAUR6326-142:YFP:HA protein, and the lower band is a presumed smaller breakdown product. In genotype labels, S63 denotes SAUR63, letters and numbers after genotype names indicate distinct transgenic lines, FL denotes a strong full-length P35S:SAUR63:YFP:HA line used as a common standard line in most experiments, and wt indicates wild-type Columbia lacking any transgene. (TIF) [file pgen.1010375.s014.tif]

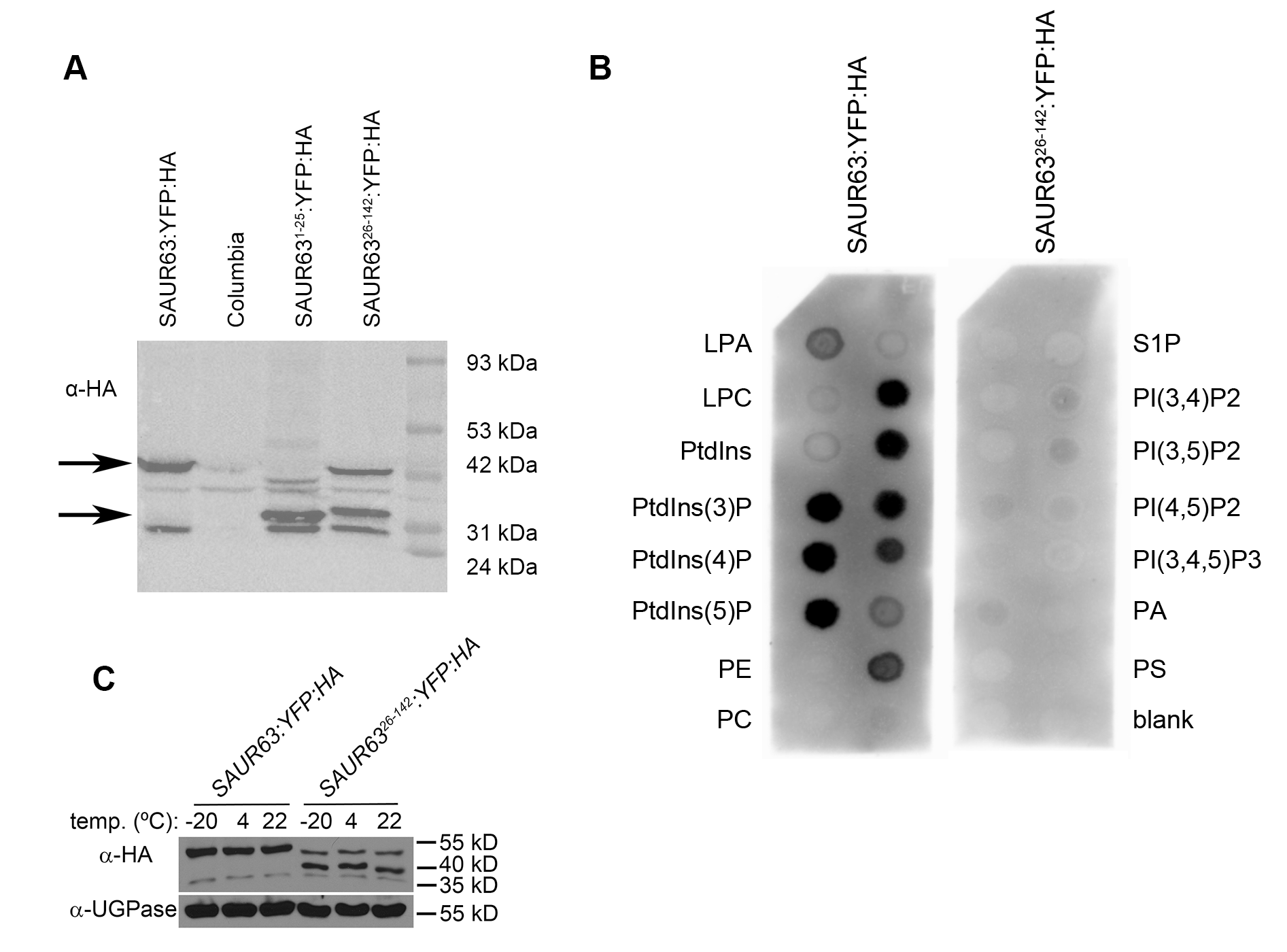

Supplement: S10 Fig — A) Western blot showing presence of fusion proteins in extracts used in lipid blot experiments in Fig 5A. Arrows indicate locations of full-length SAUR63:YFP:HA and truncated SAUR6326-142:YFP:HA fusion proteins (upper arrow) and SAUR631-25:YFP:HA N-terminal domain fusion protein (lower arrow). B) Longer exposures of two lipid blots from Fig 5A. C) Mock experiment in which extracts were incubated for 70 minutes in protein extraction buffer at the indicated temperatures, and then run on a gel for western blots. For both SAUR63:YFP:HA and SAUR6326-142:YFP:HA, similar amounts of protein are present after incubation at -20 C or after incubation at 22 C, as during the lipid blot binding experiment. (TIF) [file pgen.1010375.s015.tif]

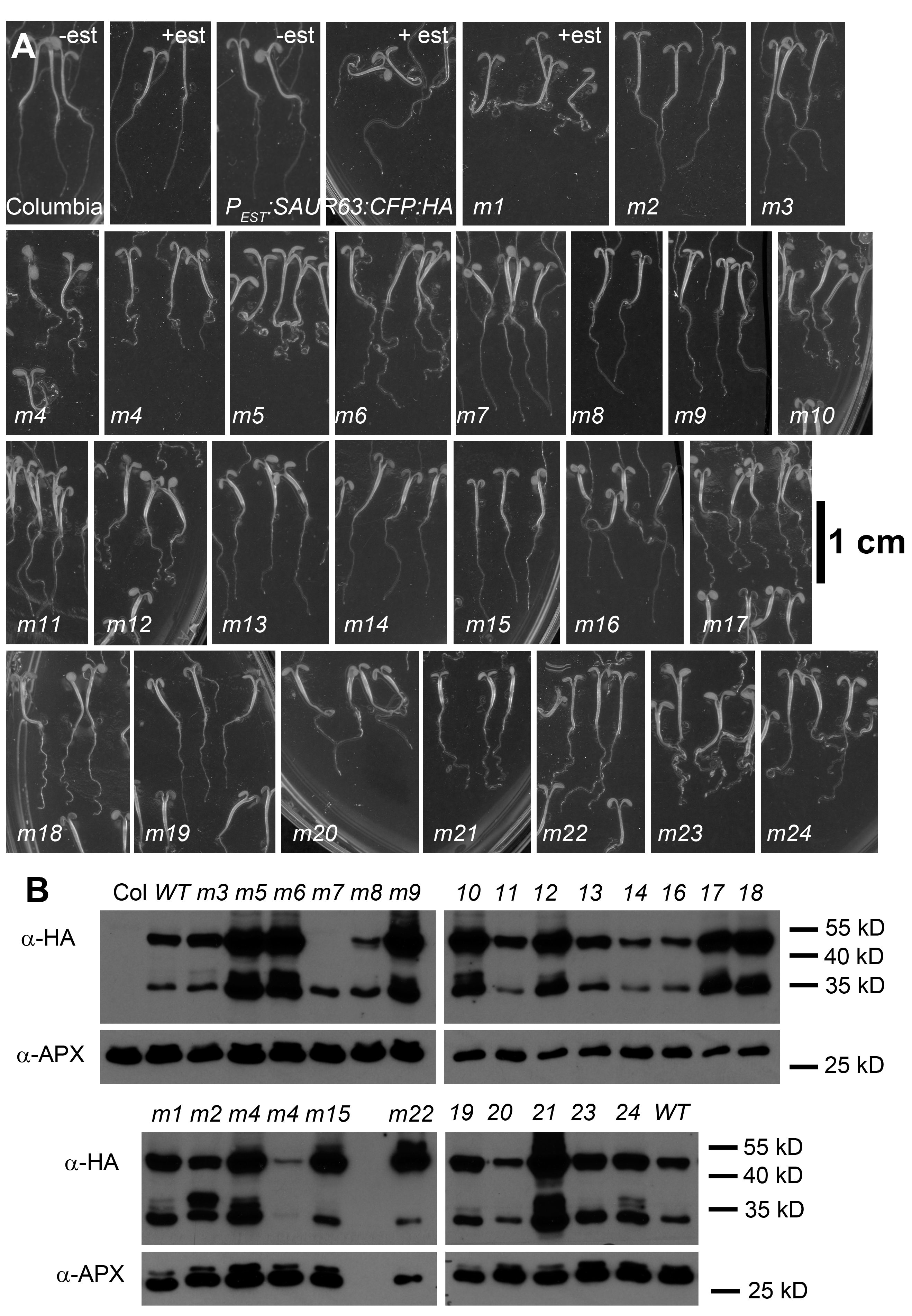

Supplement: S11 Fig — A) PEST:SAUR63NAAIRS:CerFP:HA lines grown on plates with estradiol (all genotypes) or without estradiol (wild-type Columbia and PEST:SAUR63:CerFP:HA only). S2 Table indicates amino acids changed in each mutant and root tortuosity index measurement data from this and one replicate experiment. Scale bar, 1 cm. B) Western blots of total protein in estradiol-induced PEST:SAUR63NAAIRS:CerFP:HA lines using α-HA antibody. (TIF) [file pgen.1010375.s016.tif]

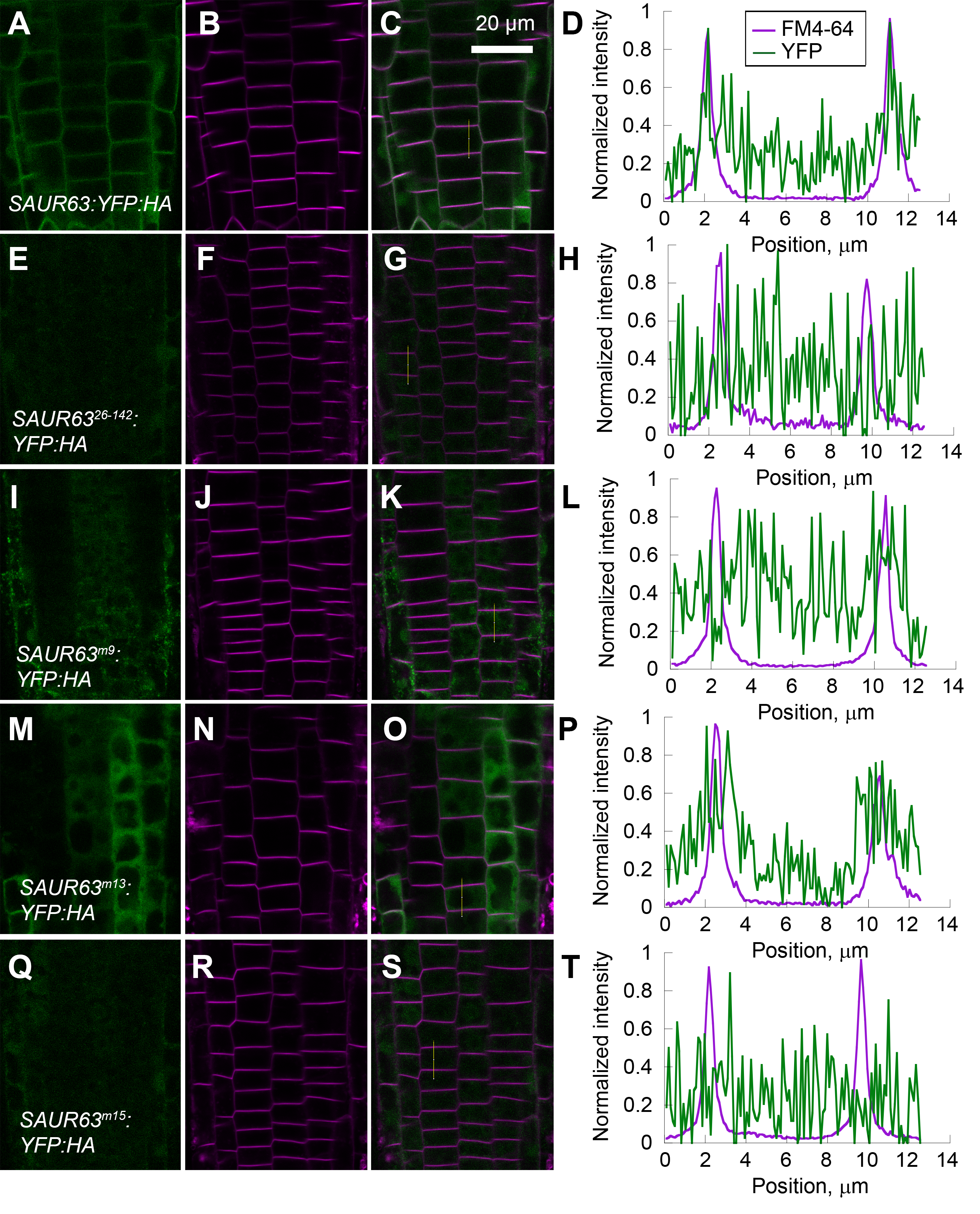

Supplement: S12 Fig — A-D) P35S:SAUR63:YFP:HA. E-H) P35S:SAUR6326-142:YFP:HA. I-L) P35S:SAUR63m9:YFP:HA. M-P) P35S:SAUR63m13:YFP:HA. Q-T) P35S:SAUR63m15:YFP:HA. Shown are fluorescence confocal microscopy images of YFP (green, A,E,I,M,Q), FM4-64 membrane staining (magenta, B,F,J,N,R), and both channels together (C,G,K,O,S) with vertical yellow lines indicating locations of quantitation of fluorescence intensity signals, scaled to the maximum signal along the line (D,H,L,P,T). Image color channel brightnesses were adjusted for visibility. Panels A-D are the same as in Fig 8. Scale bar, 20 μm. (TIF) [file pgen.1010375.s017.tif]
